# Phylogenetic inference of the emergence of sequence modules and protein-protein interactions in the ADAMTS-TSL family

**DOI:** 10.1371/journal.pcbi.1011404

**Published:** 2023-08-31

**Authors:** Olivier Dennler, François Coste, Samuel Blanquart, Catherine Belleannée, Nathalie Théret

**Affiliations:** 1 Univ Rennes, Inria, CNRS, IRISA, UMR 6074, Rennes, France; 2 Univ Rennes, Inserm, EHESP, Irset, UMR S1085, Rennes, France; Robert Koch Institute: Robert Koch Institut, GERMANY

## Abstract

Numerous computational methods based on sequences or structures have been developed for the characterization of protein function, but they are still unsatisfactory to deal with the multiple functions of multi-domain protein families. Here we propose an original approach based on 1) the detection of conserved sequence modules using partial local multiple alignment, 2) the phylogenetic inference of species/genes/modules/functions evolutionary histories, and 3) the identification of co-appearances of modules and functions. Applying our framework to the multidomain ADAMTS-TSL family including ADAMTS (A Disintegrin-like and Metalloproteinase with ThromboSpondin motif) and ADAMTS-like proteins over nine species including human, we identify 45 sequence module signatures that are associated with the occurrence of 278 Protein-Protein Interactions in ancestral genes. Some of these signatures are supported by published experimental data and the others provide new insights (e.g. ADAMTS-5). The module signatures of ADAMTS ancestors notably highlight the dual variability of the propeptide and ancillary regions suggesting the importance of these two regions in the specialization of ADAMTS during evolution. Our analyses further indicate convergent interactions of ADAMTS with COMP and CCN2 proteins. Overall, our study provides 186 sequence module signatures that discriminate distinct subgroups of ADAMTS and ADAMTSL and that may result from selective pressures on novel functions and phenotypes.

## Author summary

The vast majority of proteins have a modular multidomain organization. Domains are conserved building blocks of proteins that are widely used to characterize and predict protein functions. However, the organization of multidomain proteins underlies a great complexity and their biological role is generally not the sum of the functions attributed to each domain. To address this problem, it is necessary to develop new methods to better identify functional signatures. Here, we developed a framework based on partial local multiple alignments (to find conserved sequence modules) and phylogenetic reconciliation methods (to integrate the evolution of species, genes, sequence modules, and Protein-Protein Interactions). Application of our framework to the search for functional sequence modules in extracellular matrix proteins from the ADAMTS (A Disintegrin-like and Metalloproteinase with ThromboSpondin motif) and ADAMTSL (ADAMTS-like) family, enabled to highlight sequence signatures potentially involved in Protein-Protein Interactions.

## Introduction

**Fig 1:**
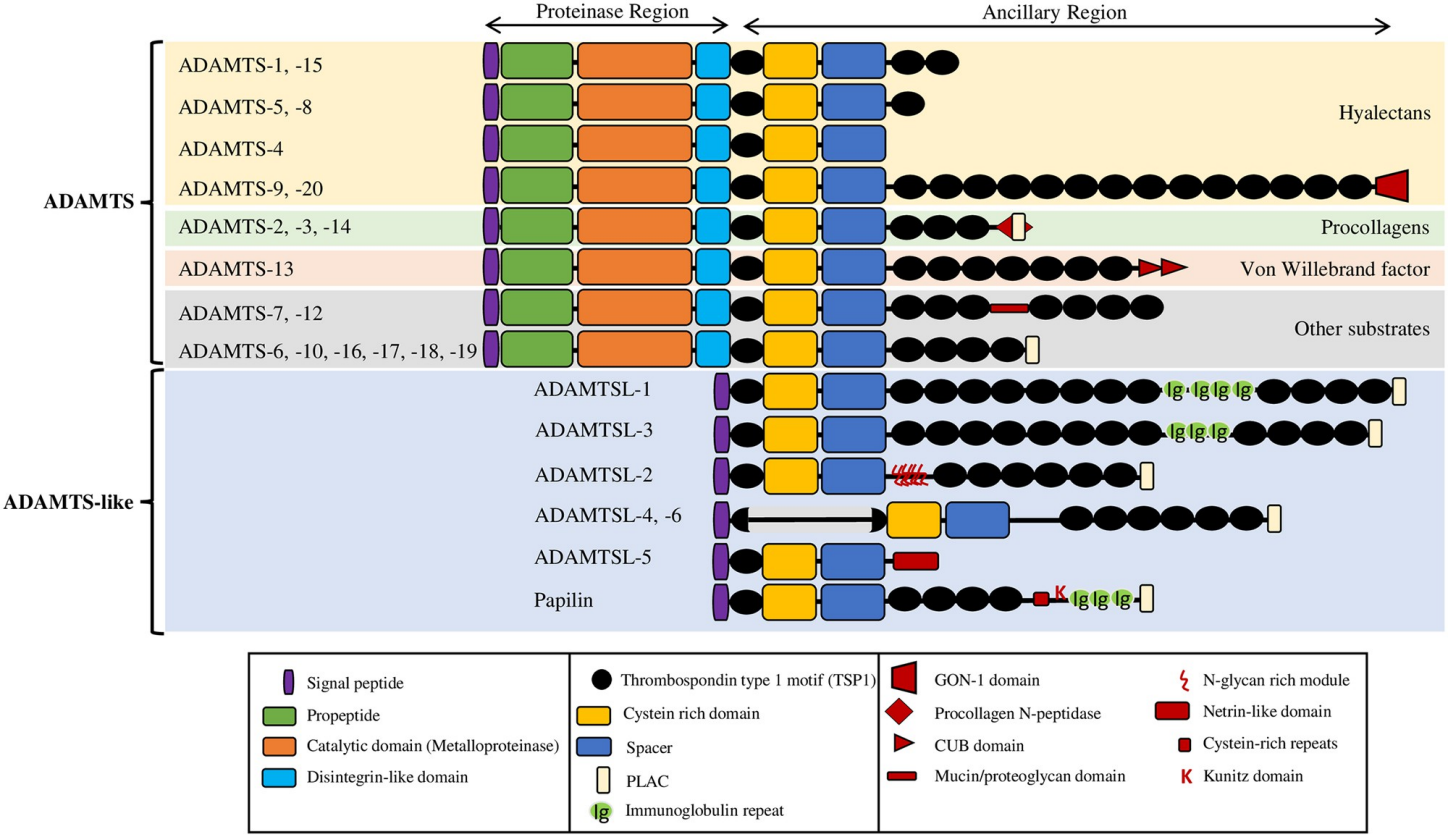
Domain/motif organization of the 26 human ADAMTS-TSL paralogs, adapted from [5].

The ADAMTS (for “a disintegrin-like and metalloproteinase with thrombospondin motif”) and ADAMTSL (for ADAMTS-like) are secreted proteins involved in extracellular matrix remodelling. They are associated with a wide range of human diseases, including cancer, fibrosis, arthritis, and cardiovascular disease [1, 2]. These homologous multidomain proteins are present in all metazoan species. The number of paralogs varies considerably from 4 genes in *C. elegans* to 26 genes in *H. sapiens*, suggesting that the ADAMTS-TSL protein family undergoes a large expansion in the number of paralogs during metazoan evolution. ADAMTS-TSL proteins are usually described according to their domain/motif composition as shown for the human paralogs in Fig 1. ADAMTS sequences are composed of two regions. The proteinase region includes a signal peptide, a pro-peptide, a catalytic domain (metalloproteinase) and a disintegrin domain. The ancillary region contains a cystein-rich domain, a spacer region, a variable number of thrombospondin type 1 motifs (TSP1) and additional domains/motifs. Until now, ADAMTS proteins have been mainly studied as proteolytic enzymes and classified according to their substrates: the procollagenase (ADAMTS-2, -3, -14), the hyalectanases (ADAMTS-1, -4, -5, -8, -9, -15, -20) and ADAMTS-13 which specifically cleaves the von Willebrand factor. The remaining ADAMTS, ADAMTS-6, -7, -10, -12, -16, -17, -18, -19, have long been considered as orphan enzymes, however following the identification of numerous substrates over the last decade they have been grouped as fibrillin/fibronectin-associated ADAMTS proteases [3]. Unlike ADAMTS, ADAMTS-like proteins lack the proteinase region but their ancillary region is similar, sharing notably the first TSP1 motif, the cystein-rich domain and the spacer region. Both ADAMTS and ADAMTSL contribute to microfibril formation through interaction with matrix components [4].

Because of their complex structure and their biochemical properties, the functional characterization of ADAMTS-TSL using classical experimental approaches remains a challenge [6]. In addition, performing specific predictions using computational methods is complicated by the lack of structural data, the complex multidomain organization of these proteins and the presence of paralogs. To date, functional predictions mainly consist in recognizing known conserved domains/motifs thanks to pre-computed signatures available in databases such as PROSITE [7] or Pfam [8]. While this approach provides a first approximation of ADAMTS-TSL functions, it fails to account for specific activities. For instance, the closely related paralogs ADAMTS-8 and ADAMTS-5 share the same domain organization but ADAMTS-5 cleaves aggrecan/versican highly efficiently [9–11] while ADAMTS-8 does not [12]. These observations suggest that more refined methods are needed to identify the specific sequence regions associated with the functional specificity of ADAMTS-TSL proteins. Instead of relying on external generic domains/motifs, we propose here to consider all local conservation of sequences within the ADAMTS-TSL family as potential evolutionary traces of their functional significance. The detection of all the conserved loci from at least two proteins of the family is made possible by partial and local multiple alignment of their sequences. Introduced by the Protomata suite [13, 14], this kind of alignment is composed of sets of local alignments involving each a subset of the sequences to align. These local alignments enable the identification of all the conserved *sequence modules* maintained during evolution, at least in a subset of sequences, by selective pressures, and thus likely to be important functionally. To identify which of these modules are associated with specific functions, we propose here to use phylogenetic inference methods to reconstruct ancestral histories of both the modules and the functions, and to predict their co-appearance along the ADAMTS-TSL evolutionary tree. The consideration of both sequence similarities and the evolutionary history of genes to propagate functional annotations along their phylogenetic tree was pioneered by Eisen et al. [15]. Subsequent studies focused on the inference of ancestral functions from the known functions in the extant genes, and more elaborated parsimonious, Bayesian or maximum likelihood evolutionary models of functional change over time have been proposed since [16–21]. These models assume that ancestral traits are likely to be shared by descendants and allow the reconstruction of an evolutionary scenario of acquisition and loss of function from scattered observations. Recently, phylogenetic methods have been proposed to estimate multi-paralog and multi-domain gene family histories [22–24]. These methods infer the so called *reconciliation* of the species, gene and domain phylogenies and enable to estimate ancestral domain compositions.

Here we present an innovative approach that relies on conserved sequence modules instead of domains. We apply the above mentioned methods to estimate the co-appearance of functional traits, i.e. Protein-Protein Interactions (PPIs), with conserved sequence modules, given the ADAMTS-TSL gene phylogeny. In a given ADAMTS-TSL ancestor, all the gained ancestral modules form a signature that is possibly involved in a gained ancestral function. Applying this new approach to ADAMTS-TSL proteins enabled us: 1) to identify 186 sequence module signatures that discriminate distinct sub-groups of ADAMTS-TSL; 2) to infer 45 signatures associated with the emergence of 278 PPIs in 45 ADAMTS-TSL ancestors; 3) to identify non-trivial convergent interactions of ADAMTS-TSL with COMP and CCN2 proteins; 4) to observe that module changes during evolution mainly occurred in the N-terminus part and in the ancillary domain, highlighting the importance of both regions in the divergence of ADAMTS-TSL specificities, and 5) to characterize ADAMTS-5 by a sequence signature that might explain its functional specificity with respect to other hyalectanases.

## Materials and methods

**Fig 2:**
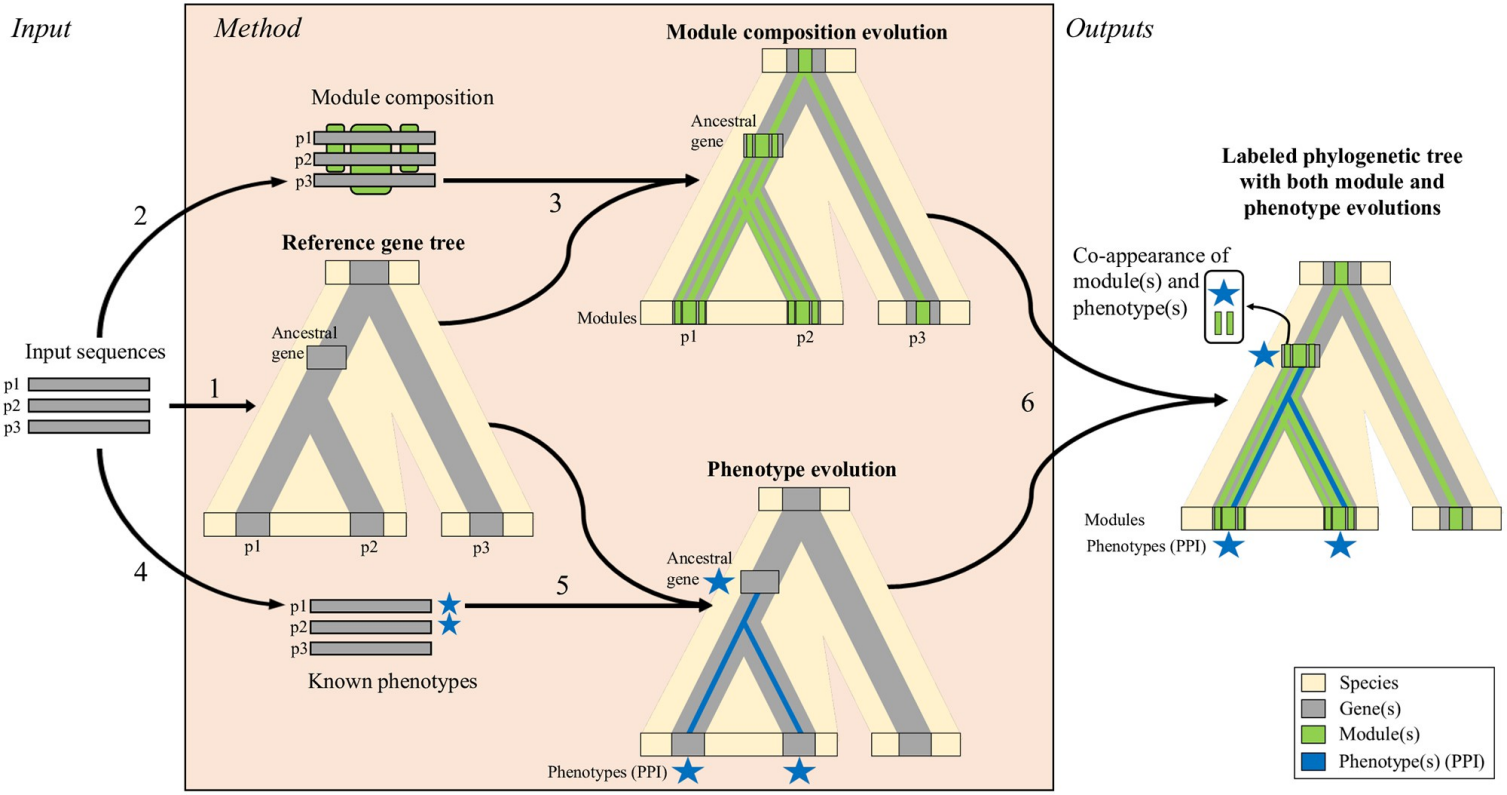
Phylogenetic inference of module and phenotype appearances. The different steps of the method, illustrated here for a dummy set of sequences containing two paralogs p1 and p2 (from one species) and their ortholog p3 (from another species), are: 1) Inference of the reference gene tree from protein sequences by a standard pipeline (*PASTA, RAxML, TreeFix*); 2) Identification of conserved sequence modules (i.e. sets of strongly similar segments from at least 2 protein sequences aligned in PLMA blocks by *Paloma-D*); 3) Inference of the module composition of ancestral genes in the reference tree (through Module-Gene-Species reconciliation by *SEADOG-MD* using the phylogenetic tree of each module inferred with *PhyML* and *TreeFix*); 4) Annotation of proteins with known phenotypic traits of interest (here Protein-Protein Interactions); 5) Reconstruction of the ancestral scenario of phenotype evolution across the reference gene tree (*PastML*); 6) Merging module and phenotype evolutionary information: each ancestral gene of the reference gene tree is then characterized by a module composition and a set of phenotypic traits (protein interactants here). The final result is the prediction of functional signatures by identification of module(s) and phenotypic trait(s) co-appearance.

The general workflow of our new approach is summarized in Fig 2. We detail in this section the different steps for identifying functional sequence modules in ADAMTS-TSL proteins.

### Species selection and sequence retrieval

To build the set of input sequences, we selected 9 species: *Homo sapiens, Mus musculus, Bos taurus, Gallus gallus, Xenopus tropicalis, Danio rerio, Ciona intestinalis, Caenorhabditis elegans,* and *Drosophila melanogaster*, because they are representative of the evolutionary history of the ADAMTS-TSL family from the bilateria ancestor to *Homo sapiens* and they have complete proteome and genome annotations in the Gene Feature Format (GFF) datasets (S1 Table). Providing the whole proteomes of the nine species as input, we used the *Orthofinder* software [25] (v2.5.2, default settings) to cluster the sequences into 31,229 *orthogroups*. An orthogroup is defined as a group of sequences from different species descending from a common ancestor gene. We identified 22 orthogroups containing at least one of the 159 RefSeq sequences of known human ADAMTS (102) and ADAMTSL (57) proteins, 17 orthogroups corresponding to ADAMTS proteins and 5 to ADAMTSL proteins. We also selected the 2 orthogroups corresponding to 7 RefSeq sequences of 4 human ADAM genes to use them as an outgroup enabling to root the ADAMTS-TSL phylogeny. Like ADAMTS-TSL, the ADAM (for A Disintegrin And Metalloprotease) proteins belong to the adamalysin family. The resulting dataset, called dataset-708, is composed of 708 protein sequences (386 ADAMTS, 226 ADAMTSL, 96 ADAM) from 24 selected orthogroups and is detailed in Table 1.

**Table 1:** Distribution of protein sequences among the nine species. The dataset-708 contains all sequences of the 24 selected orthogroups (several isoforms for a single gene). The dataset-214 contains one representative sequence per gene (described in S3 Table).

Orthogroups	ADAMTS	ADAMTSL	Outgroup	Sum				
	17	5	2	24				
Species	Dataset-708				Dataset-214			
	ADAMTS	ADAMTSL	Outgroup	Sum	ADAMTS	ADAMTSL	Outgroup	Sum
*Homo sapiens*	102	68	20	190	19	7	4	30
*Mus musculus*	106	37	11	154	19	7	4	30
*Bos taurus*	42	32	7	81	19	7	4	30
*Gallus gallus*	55	38	17	110	18	6	5	29
*Xenopus tropicalis*	31	16	9	56	19	8	6	33
*Danio rerio*	34	19	14	67	22	7	8	37
*Ciona intestinalis*	9	11	5	25	6	4	4	14
*Drosophila melanogaster*	5	3	9	17	2	1	3	6
*Caenorhabditis elegans*	2	2	4	8	1	1	3	5
Sum	386	226	96	708	125	48	41	214

The orthogroups can include several isoforms from a single gene, however functional annotations are usually available at the gene level only. We thus chose to filter out isoforms in order to select one representative protein sequence per gene per species. In a given species, the products of a gene can be defined as a group of isoforms whose loci overlap on the genome (*i.e.* they share exons). Each protein sequence is associated with its locus according to its coordinates as described in the GFF file. In a species, two protein loci overlapping by at least one residue on the same genome strand were considered as two isoforms encoded by the same gene. The longest isoform sequence of a gene was then selected as the representative protein without significant loss of information as shown in S2 Table. The resulting dataset, called dataset-214, contains 214 representative proteins from 214 distinct genes over the 9 species: 173 ADAMTS-TSL (125 ADAMTS, 48 ADAMTSL) plus 41 ADAM. All the 26 *H. sapiens* ADAMTS-TSL genes are represented in the dataset (see Table 1).

### Inference of the reference gene tree (step 1)

The gene phylogeny, starting point of our method, was inferred from the 214 representative protein sequences in dataset-214.

As a first step, we used the multiple sequence aligner PASTA [26] (v1.8.5, default settings) to compute the multiple alignment of the 214 proteins and the maximum likelihood phylogeny inference software RAxML [27] (version provided with PASTA, default settings) to infer a first initial phylogeny. This initial tree was corrected using *TreeFix* [28] (v1.1.10, 100 iterations with the PROTGAMMAJTT model) taking into account the species tree defined in the NCBI taxonomy, thus possibly changing the topology of the initial tree. The branch lengths (lost by TreeFix) were then recomputed using *PhyML* [29] (v3.3.20190909, optimizing only branch lengths and substitution rate parameters with amino acid default settings) and the tree was rooted using ADAM proteins as the outgroup, resulting in the final phylogeny that we named the *reference gene tree*. To analyse the uncertainty resulting from the taxon and gene sampling, and to identify robust bi-partitions in the reference gene tree, we analyzed several ADAMTS-TSL protein samples by varying the content of the outgroup and by adding ADAMTS-TSL sequences of species not considered in our 9 species (see S1 Appendix).

### Identification of conserved sequence modules (step 2)

The strongly conserved sequence modules in ADAMTS-TSL proteins were identified by *partial local multiple alignment* of the 214 sequences in dataset-214.

**Fig 3:**
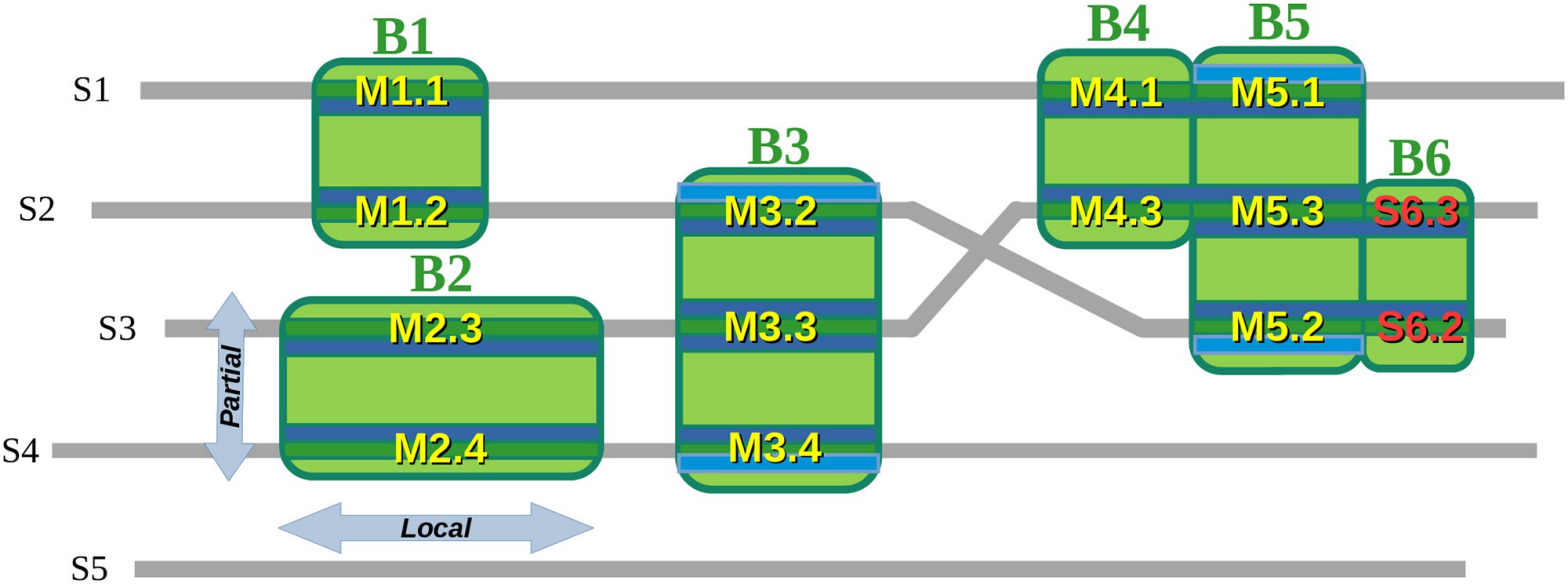
Identification of modules by partial local multiple alignment. We show here a schematic PLMA of sequences *S*1, …, *S*5 composed of the alignment blocks *B*1, …, *B*6. This example illustrates the locality of the alignments: each alignment of positions is supported by a local alignment of sequences, leaving possibly other sequence positions unaligned. For instance the alignment block *B*2 is local since it aligns only the two segments *M*2.3 and *M*2.4 of the sequences *S*3 and *S*4, and no other positions of these sequences. *Local* alignments authorize to align only *subsets of adjacent positions* from each sequence. In an orthogonal way, *partial* alignments authorize to align positions of only a *subset of the sequences*. This is also illustrated by *B*2 which is partial since it aligns only positions from *S*3 and *S*4 (and does not even align them to positions of the block *B*1 above *B*2). Partial local alignments are not limited to pairwise alignment: the block *B*3 is an example of partial local alignment block, aligning the segments *M*3.2, *M*3.3 and *M*3.4 from sequences *S*2, *S*3 and *S*4, that can be built from the pairwise local alignments (highlighted here in blue colors) of the segments *M*3.2 with *M*3.3, *M*3.3 with *M*3.4 and *M*3.2 with *M*3.4. The PLMA blocks align each a set of *segments* conserved specifically in a sequence subset. This set of segments, provided that they are long enough, is said to be a conserved sequence *module*. Let us assume here that all the blocks, except *B*6, align segments of 5 or more residues. The set of segments {*M*2.3, *M*2.4} aligned in *B*2 defines then for instance the module *M*2, while the block *B*3 enables to identify the module *M*3 = {*M*3.2, *M*3.3, *M*3.4}. Blocks *B*4, *B*5 and *B*6 illustrates how the definition of the blocks –requiring that each segment is aligned to, and only to, all the other segments of the block– enables to split possibly longer local alignments to identify segments specifically conserved in sequence subsets: even if the concatenation of *M*4.1 and *M*5.1 is locally aligned to the concatenation of *M*4.3 and *M*5.3 (this could be for instance a pairwise alignment used to build the PLMA), none of these two concatenations is locally aligned to *M*5.2. In this case, the maximal set of segments aligned with *M*5.2 is {*M*5.1, *M*5.3, *M*5.2} and the modules are here *M*4 = {*M*4.1, *M*4.3} specifically conserved in {*S*1, *S*3} and *M*5 = {*M*5.1, *M*5.3, *M*5.2} specifically conserved in {*S*1, *S*3, *S*2}. Similarly, the block *B*6 aligns the segments *S*6.3 and *S*6.2 but, in contrast with the segments aligned by *B*4, these segments are shorter than 5 residues and do not define a module.

Introduced by the Protomata suite [13, 14], a partial local multiple alignment (PLMA) consists of *a set of alignment blocks* containing each a set of locally aligned segments from some, but not necessarily all, of the given sequences (as in partial order alignments [30], but with the addition of a locality property, see Fig 3). The Protomata suite provides the Paloma program to build a PLMA by iterative selection and integration of *pairwise local alignments* under compatibility constraints. To efficiently process the large number of long and similar sequences available, we used Paloma-D (v0.1), a new and faster re-implementation in modern C++ of Paloma (see S2 Appendix).

The PLMA of the 214 sequences contained in the representatives.fasta file was computed with the command line paloma-d -i representatives.fasta -M 20 -t 10. The alignment procedure is detailed in S2 Appendix. With these parameters, the candidate pairwise local alignments were all the *diagonals* (i.e. gap-free pairwise local alignments) computed by the program dialign2–2 [31], of length smaller or equal to 20 residues and with a strong similarity weight of 10 or more (corresponding to similarities of probabilities lower than 5 × 10^−5^ in random sequences). The multiple sequence alignment was built from this set of pairwise alignments of interest as follows. First, the candidate diagonals were sorted by the default heuristic of Paloma-D, putting forward those with the stronger evidence of conservation across all the sequences. Then, they were iteratively added in this order to the PLMA, with transitive alignment and local extension of diagonals, if and only if the consistency of the resulting induced alignment [32] was preserved.

The conserved sequence modules were identified from the alignment blocks of the resulting PLMA. Alignment blocks are the maximal alignment of segment units verifying that each segment of a block is aligned by the PLMA over its full length to, and only to, all the other segments of the block. Let us remark that block segments can thus be shorter than the pairwise local alignments used to build the PLMA. With the chosen parameters, each PLMA block aligned without gaps a set of segments of same length which were strongly conserved in one, and only this, subset of the 214 sequences. We defined the conserved sequence *modules* to be all the *sets of segments* that were aligned by these PLMA blocks, provided that their length was greater than 5 residues (see Fig 3). The minimal length of 5 residues was required here to enable us inferring the module’s phylogenies in the following step.

### Inference of the module composition of ancestral genes (step 3)

The step 3 consists in building the evolutionary history of the modules, according to the reference gene tree.

First, the phylogenetic tree of each module was inferred from the gapless alignment of its segments (as in the corresponding alignment blocks from step 2) by Maximum Likelihood software *PhyML* [29] (v3.3.20190909, amino acid default settings). Then, each module tree was corrected by *TreeFix* [28] (v1.1.10, 100 iterations with the PROTGAMMAJTT model) with respect to the reference gene tree. Of note, the final topology of the module tree might still be different and incompatible with the one of the reference gene tree. Then the module tree was mapped on the reference gene tree using the phylogenetic reconciliation software *SEADOG-MD* [24] (default options) with the help of the species tree (the evolutionary tree according to NCBI Taxonomy [33]). This resulted in mapping each internal node of the module tree to an internal node *n* of the reference gene tree, enabling us to infer an occurrence of an ancestral segment of this module at the ancestral gene *n*.

Mapping all the modules, each ancestral gene node *n* of the reference tree was assigned an ancestral module composition $M_{n}$, so the reference gene tree is annotated with the module composition. It represents the inferred ancestral evolution of module composition.

### Collecting Protein-Protein Interactions as phenotypic traits of interest (step 4)

The step 4 consists in collecting functional traits associated with sequences.

Because of their importance in the functions of proteins, we chose Protein-Protein Interactions (PPIs) as the phenotypic trait of interest. The conversion of the RefSeq ID of the 386 ADAMTS and 226 ADAMTSL sequences from dataset-708 enabled us to retrieve 494 UniprotKB ID that were used to query molecular interaction databases using the Proteomics Standard Initiative Common QUery InterfaCe (PSICQUIC) [34]. The retrieved file contained 1985 lines (S4 Table) including interactions between proteins of the same species (e.g. 1685 for *H. sapiens*, 28 for *M. musculus* and 106 for *C. elegans*) and interactions between proteins from different species (e.g. 65 for *H. sapiens*/*M. musculus*). The 66 interactions involving ADAMTS-TSL and complexes are excluded because of the lack of protein identification from complexes. After filtering redundant identifiers, 461 different PPIs were annotated (S5 Table). They included 395, 13 and 36 PPIs for *H. sapiens*, *M. musculus* and *C. elegans*, respectively. Analysis of the data in mice unsurprisingly shows redundancy with humans. Most *C. elegans* PPIs (32/36) involve papilin (mig-6) and the remaining four PPIs involve madd-4 (orthologs of ADAMTSL1 and 3) and adt-2 (ortholog of ADAMTS2), but there is no PPI shared with human. In addition, 8 interactions between human and mouse proteins are redundant with each species and the remaining 8 PPIs involve human proteins and proteins from other organisms such as viruses and bacilli. In conclusion, sequence queries of the nine species yielded very little information about the non-human species and we chose to retain only the PPIs from *H. sapiens* in the PSICQUIC results. Importantly, we observed that many recently identified substrates of ADAMTS were not described in molecular interaction databases and we decided to complete the list of PPIs by manual curation of the literature. Our review of the literature enabled us to identify an additional set of 303 PPIs (S6 Table). In this dataset, we included high throughput data from the iTRAQ-TAILS method that was used to identify novel substrates for the procollagenases ADAMTS2,-3 and -4 [35, 36] and the ADAMTS7 protein [37]. We also included four PPIs involving LRP1 which is not a substrate but interacts with ADAMTS4,-5,-9,-10 to mediate ADAMTS internalization and thus regulate their activities [38, 39]. Merging data from PSICQUIC queries and manual curation resulted in a PPI_human_ set of 662 non-redundant documented interactions between one of the 26 *H. sapiens* ADAMTS-TSL representative protein and one of their 471 *H. sapiens* protein interactants (S7 Table).

### Reconstruction of the ancestral scenario of phenotype evolution (step 5)

The step 5 consists in building the evolutionary history of the PPI, according to the reference gene tree.

Ancestral ADAMTS-TSL Protein-Protein Interactions along the reference gene tree were inferred with *PastML* (v 1.9.33, default setting) [20]. Three kinds of status can be given as input to *PastML* on the ability of a protein at a leaf of the tree to interact with each partner: “able to interact” (1), “not able to interact” (0) or “missing annotation” (?); each missing annotation being later inferred by *PastML* as able to interact (1) or not able to interact (0). As stated in step 4, we focus on *H. sapiens* PPIs. We set the status of *H. sapiens* representative protein to “able to interact” (1) for each interacting partner listed in PPI_human_ and “not able to interact” (0) for the other human proteins. Given the very limited amount of information on PPIs in non-human species, we chose to set the status at the leaves to “missing annotation” (?) for each PPI.

Given these leaf annotations and the reference gene tree, *PastML* associated to each node *n* (internal or leaf) with missing annotation a set of interactants $I_{n}$. Let us note that *PastML* proposes different coherent evolution scenarios based on marginal probabilities of character (PPI presence in this study) that were also recorded. Here we focus only on the proposed most likely scenario.

The reference gene tree annotated with ancestral interactants at each node represents the inferred scenario of evolution of ADAMTS-TSL interactions with proteins.

### Prediction of functional signatures (step 6)

By integrating the module compositions (from step 3) and ADAMTS-TSL PPI annotations (from steps 5) onto the reference gene tree, we can simultaneously examine the inferred evolutionary histories of genes, module compositions and ADAMTS-TSL protein interactions.

To focus on changes across the evolution of ADAMTS-TSL genes, we computed for each node *n* of the reference tree, the sets $M_{n}^{+}$ and $M_{n}^{-}$ of the gained and lost modules of the gene at *n* with respect to its ancestor at node *a*. A module was added to $M_{n}^{+}$ if it was in $M_{n}$ and not in $M_{a}$. Conversely, it was added to $M_{n}^{-}$ if it was in $M_{a}$ and not in $M_{n}$. The sets $I_{n}^{+}$ and $I_{n}^{-}$ of gained and lost PPI of the gene at node *n* were computed similarly from I.

The set of modules gained at a node *n*, $M_{n}^{+}$, was said to define the *module signature* of the node. The more significant module signatures are those co-appearing with a phenotypic trait at a node. Then we focused on the co-appearance of protein interactions and modules, i.e. on nodes *n* such that $M_{n}^{+}$ and $I_{n}^{+}$ were both non-empty. In that case, the set of modules $M_{n}^{+}$ was identified as a candidate functional module signature of the interaction of the ADAMTS-TSL with the members of $I_{n}^{+}$. Note that both modules and interactions are shared by some, but not necessarily all, the descendants of the ancestral gene *n*.

### Visualization of the labeled phylogenetic tree

An important contribution of this work is the availability of all the data with an interactive tree (automatically generated at the end of the pipeline), using the Interactive Tree Of Life software, Itol [40]. The ADAMTS-TSL Itol tree is available at https://itol.embl.de/tree/13125419158431781652196295. An HTML popup window provides detailed information about each gene node, including the name of the gene, the nature of the gene (ancestor or leaf), the PPIs associated with the gene and PPI gain/loss with respect to the ancestor, the module composition of the gene and module gain/loss with respect to the ancestor. The Itol tree also provides annotations including the number, the composition and the transfer of modules, the domain composition and the presence of PPIs. In particular, each gene has its own module signature annotation in which “gained” modules are represented by green boxes on the descendants. All module signatures can be visualized within their domain contexts by first enabling the domain composition annotations. All the inferred module signatures for the ADAMTS-TSL family are available at https://github.com/OcMalde/PhyloCharMod_publ (see S3 Appendix for screenshots and examples of usage).

## Results

### Evolutionary histories of ADAMTS-TSL genes, modules, and PPIs

**Fig 4:**
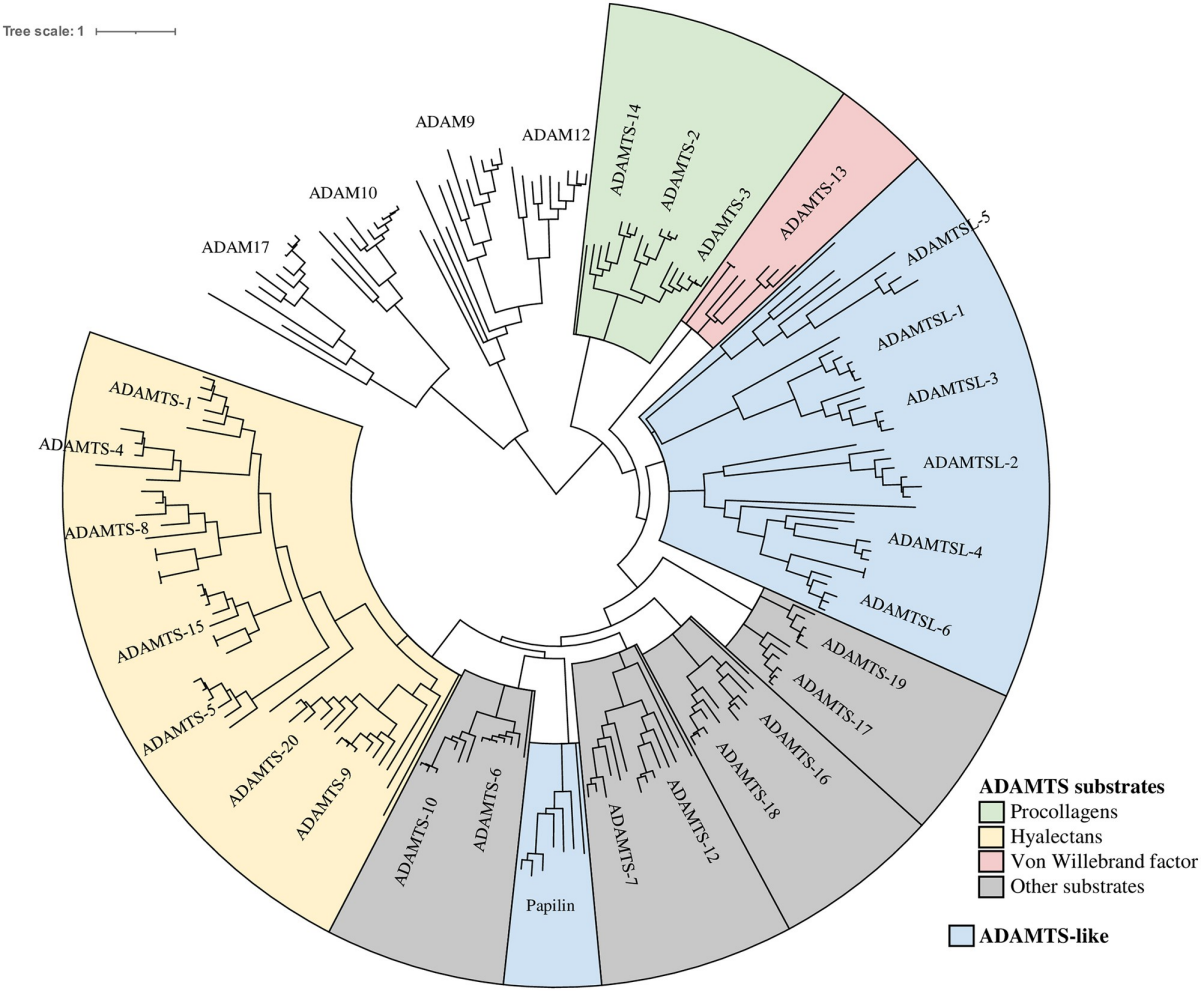
Reference gene tree of the 125 ADAMTS, 48 ADAMTSL and 41 ADAM outgroup members (figure produced with Itol).

To explore the ADAMTS-TSL evolutionary history according to their diverging module compositions and PPIs, we first inferred a phylogenetic tree from ADAMTS and ADAMTSL protein sequences and rooted the tree using some ADAM protein sequences as the out-group. This reference gene tree is shown in Fig 4. We identified seven monophylies including the procollagen peptidases (ADAMTS-2, ADAMTS-3, ADAMTS-14), the hyalectanases (ADAMTS-1, ADAMTS-4, ADAMTS-8, ADAMTS-15, ADAMTS-5, ADAMTS-20, ADAMTS-9) and four groups associating pairs of ADAMTS, ADAMTS-7 and ADAMTS-12, ADAMTS-6 and ADAMTS-10, ADAMTS-16 and ADAMTS-18, ADAMTS-17 and ADAMTS-19. Note that the ADAMTSL form a separated clade that does not include papilin proteins.

**Fig 5:**
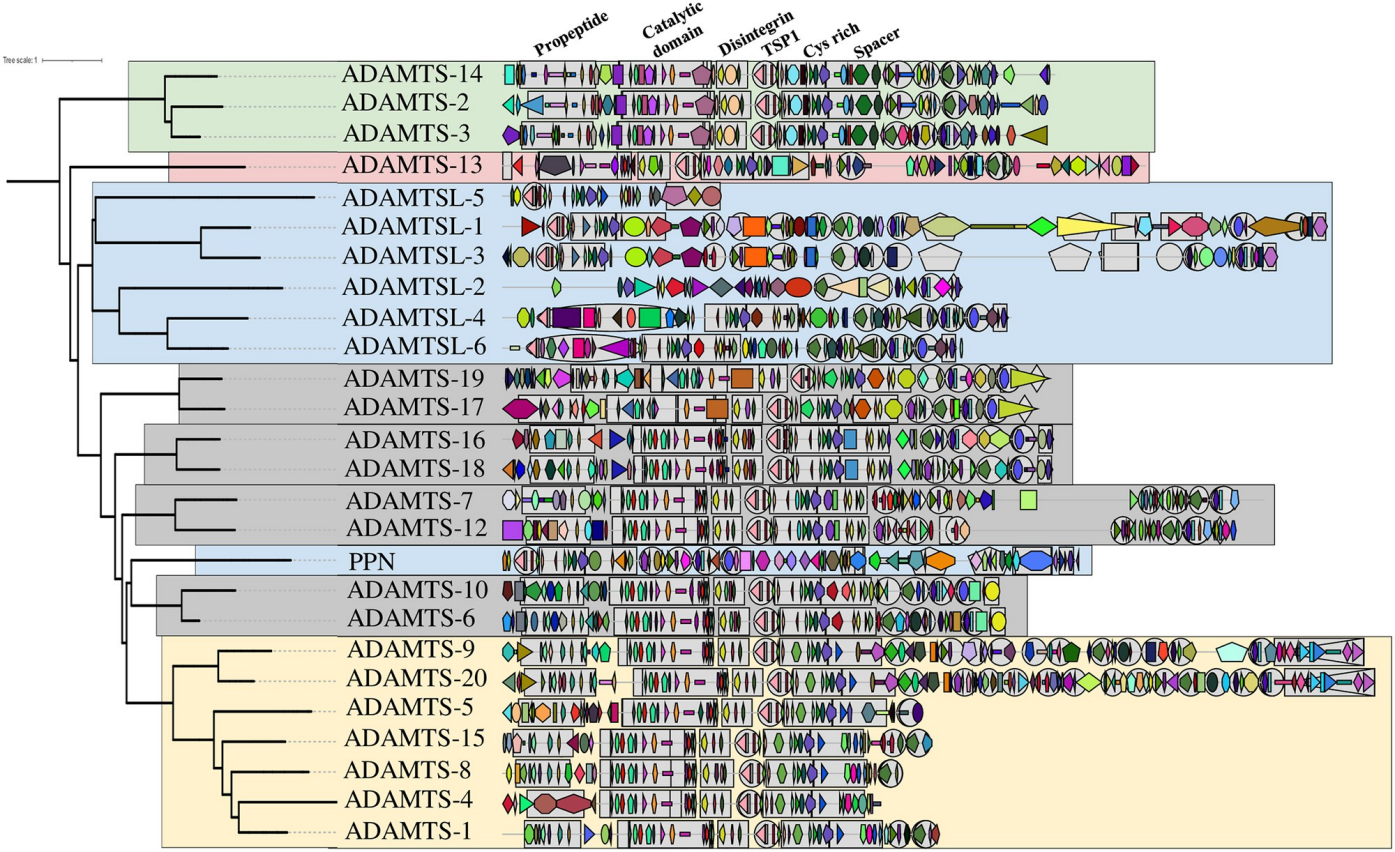
Module composition of the 26 *H. sapiens* ADAMTS-TSL sequences. The phylogenetic gene tree of the 26 *H. sapiens* ADAMTS and ADAMTSL paralogs was extracted from the reference gene tree (Fig 4). The modules identified by the Paloma-D program are represented on the sequences with Itol, using a unique combination of form and color to designate each module. The complete list of modules is provided in the S8 Table.

We then inferred the evolutionary history of the gene module composition. Using Paloma-D, the partial local alignment of the 214 sequences identified 1059 conserved sequence modules (S9 Table). As expected, some of these conserved sequence modules are shared by all sequences, while others are shared by different subsets of sequences. In particular, we identified two modules (covering a total length of 33 amino-acids) that were shared by all 26 human ADAMTS-TSL sequences and localized in the cysteine-rich and spacer domains, which are shared domains of all ADAMTS-TSL. In addition, we found 11 other modules (covering a total length of 111 amino-acids) that were specifically shared by the 19 human ADAMTS sequences, located in the catalytic domain, disintegrin, central TSP1, and cysteine-rich domains. These domains were already identified as shared among ADAMTS, which support our Paloma-D module decomposition at the ADAMTS-TSL family level. The module composition of the 26 *H. sapiens* ADAMTS-TSL sequences is detailed in Fig 5. In the inferred ancestral module composition, 186 ancestral gene nodes of the ADAMTS-TSL tree were associated with the gain of one or more modules, defining as many module signatures.

**Fig 6:**
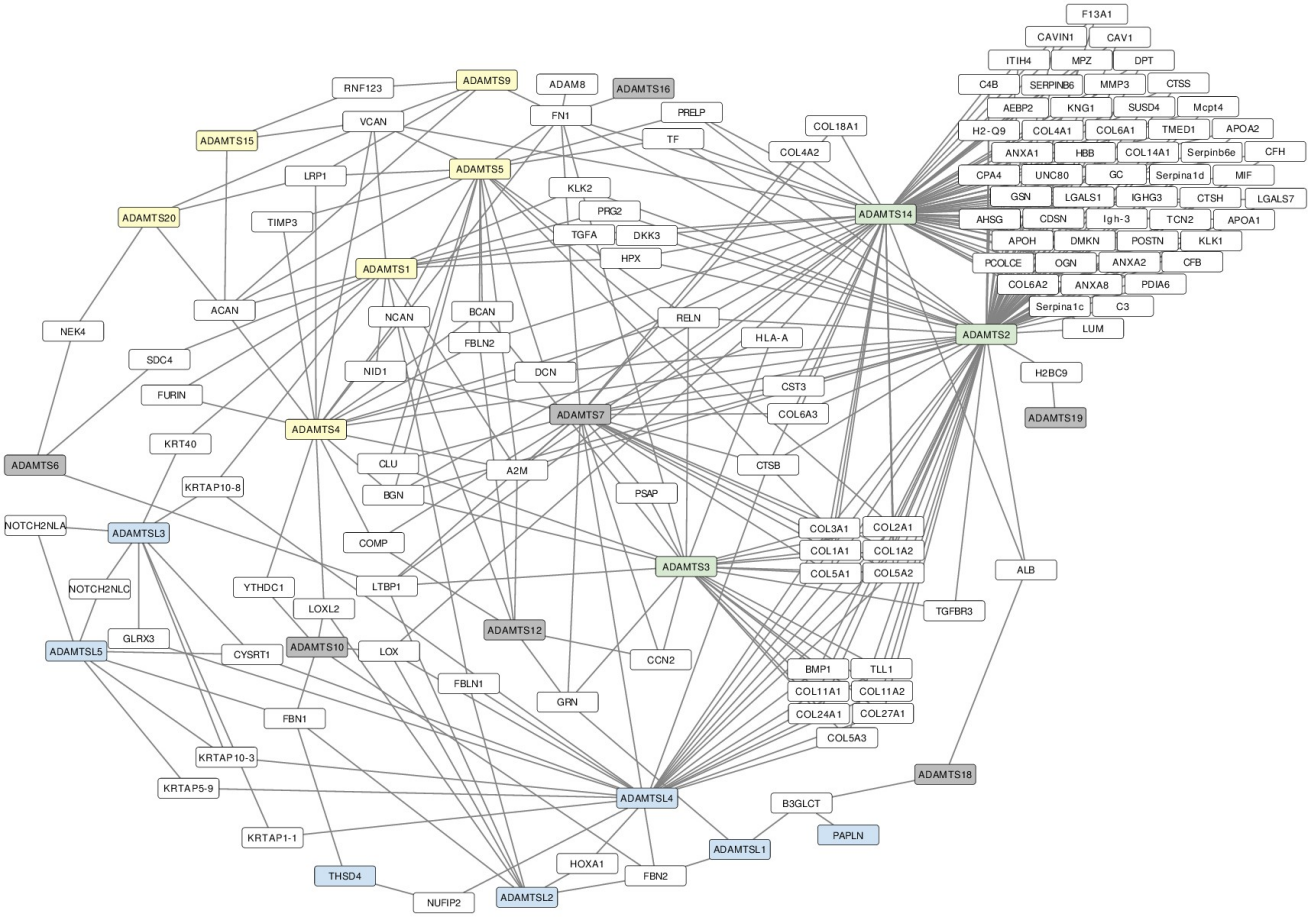
Protein-Protein Interaction networks of *H. sapiens* ADAMTS-TSL. The 119 PPIs shared by the 26 human ADAMTS-TSL are visualized with Cytoscape [41]. Yellow nodes are hyalectanases, green nodes are pro-collagenases, grey nodes are ADAMTS with unspecific substrates, blue nodes are ADAMTSL and white nodes are proteins interacting with ADAMTS-TSL.

In parallel, we investigated the evolution of PPIs involving the ADAMTS-TSL proteins and other proteins. A first result is the identification 662 PPIs involving at least one *H. sapiens* ADAMTS-TSL and one of their 471 *H. sapiens* partners. Among the 471 partners, 119 have interactions with at least 2 ADAMTS-TSL (Fig 6). Using the PastML tool, we inferred 471 ancestral scenarios representing the PPI evolution given the ADAMTS-TSL gene tree. We identified 48 ancestral genes in which one or several PPIs most likely appeared. These nodes indicate potential gain of function.

### Conserved sequence module signatures associated with PPIs

Finally, we integrated in the reference gene tree the estimated evolutionary histories of the modules and the PPIs. As a result, only 3 ancestral gene nodes (G149, G217, G329) exhibited one PPI but no module gain and 45 ancestral gene nodes exhibited both module and PPI gains (Table 2). Among these 45 ancestral gene nodes, 5 were the ancestors of at least two paralogs (nodes corresponding to paralogous duplication events: G91, G96, G161, G235 and G341), while 40 were only the ancestors of orthologs (nodes corresponding to speciation events). These 45 nodes correspond to module-PPI co-appearance events and highlight gene ancestors in which a new PPI has emerged and from which ancestor segment(s) of gained module(s) have been conserved by evolution. Based on these co-appearance events, we proposed 45 module signatures (including 355 modules) as candidates for sequence regions likely involved in 278 PPIs (as shown in Table 2). The module signatures contain from 1 to 26 modules while the PPI signatures contain from 1 to 143 PPIs. Some ancestors have few PPIs associated with few modules which make them much easier to interpret. This is the case for the ancestral gene G96 for which two PPIs (ACAN, VCAN) are associated with two modules (B773, B783). This is also the case for the ancestral gene G315 for which one PPI (CCN2) is associated with the B1424 module. In contrast, the ancestral gene G341 is characterized by the association of numerous PPIs (66) and modules (22). In addition, we observed the co-appearance of multiple modules for a single PPI such as G91 for which the interaction with LRP1 is associated with 26 modules. Note that some PPIs and modules are involved in multiple co-appearances. A possible interpretation is that these PPIs were acquired multiple times during the evolution of ADAMTS-TSL, suggesting convergent evolution.

**Table 2:** The 45 events of module(s)-PPI(s) co-appearance. G91, G96, G161, G235 and G341 are ancestral genes of at least 2 paralog gene.

Gene	Descendant(s)	PPI(s)	Module(s)
G4	ADAMTS-1	HPX DKK3	B561 B1646 B1661 B1657 B1659 B608 B1658 B605 B1668 B1647 B1645
G8	ADAMTS-1	A2M	B1673
G10	ADAMTS-1	FURIN	B687 B882 B1283 B1665 B1672 B686
G13	ADAMTS-4	RELN	B1950
G15	ADAMTS-4	A2M FN1 BGN COMP LRP1	B1693 B1675 B1678 B1497 B1674 B1496 B1692 B1676
G19	ADAMTS-4	FURIN	B1691 B1689 B1677 B1687 B1842 B1683
G23	ADAMTS-8	SPP1	B1545 B1552
G59	ADAMTS-5	NCAN CILP MATN4 FMOD SDC1 ITIH2 TNC TIMP3 MMP13 FBLN2 CILP2 ADAMTS5	B1583 B1595 B1610 B1945 B1575 B1577 B1598 B1944 B1591 B1592
G61	ADAMTS-5	DCN PRELP	B491
G63	ADAMTS-5	COL2A1 COL3A1 FN1 BGN RELN	B657 B1578 B658 B1608
G65	ADAMTS-5	A2M LRP1	B1602 B1605 B1593 B1594 B2029 B1588 B1601 B1607 B1617 B1603 B1580 B686
G71	ADAMTS-9	RNF123	B522
G75	ADAMTS-9	FN1	B974 B1734 B1714 B976 B973 B963 B983 B917 B993 B978 B1702 B873 B1708
**G91**	ADAMTS-9, -20	LRP1	B1715 B858 B949 B1958 B888 B946 B1707 B1283 B854 B836 B987 B835 B911 B860 B856 B889 B1704 B398 B913 B820 B1705 B1728 B939 B865 B910 B400
**G96**	ADAMTS-1, -4, -5, -8, -9, -15, -20	VCAN ACAN	B773 B781
G103	ADAMTS-6	ERP29 SDC4 NEK4 SNTA1 LTBP1 RNF2	B1864 B919
G114	ADAMTS-10	FBN1	B671 B676 B683 B606 B673 B702 B674
G128	Papilin	PKM THOP1 DPY19L3 KLHL36 ZNF507 TFAP2C KIF2C NIF3L1	B2223 B2233 B2265 B2253 B2222 B2214 B2268 B2257 B2241 B2240 B2255 B2251 B2228 B2254 B2232 B2252
G141	ADAMTS-12	HELS72 FKBP9 CALM2 USF1 PPM1A MEOX2 MYL6 SP3 USF2 RUFY3 UBR1 SNRK	B1318
G143	ADAMTS-12	NCAN	B1298 B1262 B687 B1249 B1320 B1296 B1287 B1290 B1286 B1299 B1253 B1300 B1260 B1291 B1325 B1336 B1245 B1297
G152	ADAMTS-7	CTSB	B560 B1919 B1918 B1917
G154	ADAMTS-7	COL5A1 COL1A2 CST3 COL1A1 COL3A1 FN1 COL5A2 COL6A3 HLAA	B1450 B1454 B1444 B1436 B1287 B1426 B1470 B1290 B1286 B1453 B1455 B1443 B1433 B1451 B1435 B1430
G160	ADAMTS-7	A2M	B1923 B1431 B1921 B1924 B1432
**G161**	ADAMTS-7, -12	GRN CCN2 COMP	B1289 B1456 B1331 B1272 B1460 B1993 B594 B592 B1305 B1429 B1283 B1332 B911 B1269 B1317 B1306 B1811 B1315 B1307 B1995 B1288 B1328 B1316 B1247 B910
G169	ADAMTS-18	B3GLCT	B1096
G171	ADAMTS-18	ALB	B477 B1073 B1095
G182	ADAMTS-16	FN1	B1025 B1012 B1057 B1893 B1004 B1003 B1894
G221	ADAMTSL-6	FBN1	B2130 B2163 B2155 B2145 B519 B2353 B2160 B2156 B2180 B2132 B2140 B509 B2124
G228	ADAMTSL-4	GLRX3 KRTAP108 KRTAP11	B2419 B2422 B2417 B2418 B2421 B2420
G230	ADAMTSL-4	EFEMP2 COL5A1 ITGB4 EGFL9 COL1A2 CLEC18A CYSRT1 US26 LCE2B PLSCR1 ADAM12 FRS3 SPRY2 PRKAB2 TLL1 MAPKBP1 SORBS3 EIF4E2 STK16 FARS2 BAG4 TOP3B COL1A1 COL2A1 COL3A1 ITGB2 COL5A2 CTSB CST2 GIP COL11A1 BMP1 COL11A2 HGF FAH VCAM1 SPINK2 FLNA GATA2 LMO2 LMO1 COL5A3 KRTAP59 COL8A1 CFP AQP1 HOXC8 OTX1 NTF4 PTGER3 HOXA1 FXR1 KRTAP101 KRTAP103 KRTAP105 KRTAP109 KRTAP1011 FKBP1B CREB5 TNK2 KIF1A PIN1 FHL3 DGCR6 DIP2A GNMT MVP TCEA2 TRIP6 LONRF1 COL24A1 KRTAP212 MGAT5B MIIP LCE3C LCE3E LCE1C LCE1B LCE4A LCE2D VASN KRTAP56 MORN3 LRFN4 SPATA8 DLK2 ADAMTSL4 ADAMTSL5 KRTAP52 KCTD9 PID1 ATG9A NEK8 TMEM150A TRIM42 CFAP206 COL27A1 NATD1 CATSPER1 DBF4B ZNF417 LGALS14 RAB2B LRRC29 MYLIP SUSD6 SMARCC1 FBXW5 DISP1 NTAQ1 OLFM3 RCHY1 TSSK3 ZNF587 CYP2S1 KRTAP412 APOL6 TAPBPL DGCR6L KRTAP94 KRTAP92 KRTAP411 KRTAP42 ASPSCR1 NMUR2 CXCL16 RHOJ BANF2 FAM124B ARNT2 SLC6A20 FBXO6 SHFL CPNE7 TUBGCP4 SLC23A1 MID2 DNPEP GNE EXOSC1 SALL2 AMMECR1 CHCHD2	B2182 B2181 B2183 B2195 B2188 B2194 B2189 B2196 B2208
**G235**	ADAMTSL-4, -6	NUFIP2	B2173 B1188 B2168 B2153 B592 B2174 B2203 B2139 B2210 B2147 B2199 B2200 B2201 B2172
G248	ADAMTSL-2	FBN1 HOXA1 LOXL3 NECAB2	B2310 B2378 B2377 B2299 B2385 B2301 B2379 B2380 B1820 B2381
G258	ADAMTSL-3	CYSRT1 GLRX3 KRTAP23 NOTCH2NLC KRTAP123 KRTAP103 KRTAP106 KRTAP108 KRTAP11 KRT40 KRTAP57 NOTCH2NLA MDFI KRTAP32 KRTAP24	B2084 B2071 B657 B2091 B2076 B2082 B2074 B519 B2102 B1287 B2079 B2042 B2391 B2072 B2395 B2394 B2078 B2087 B2393
G269	ADAMTSL-1	MMP10 FHL2 ACOX1 B3GLCT RSPRY1 WDCP	B2401 B2407 B2403 B2405 B2102 B2411 B2096 B2409
G271	ADAMTSL-1	GRN	B2105 B2114 B2098 B2107 B2113 B2110 B2106 B2109 B2112 B2104 B2108 B2344
G281	ADAMTSL-5	CYSRT1 KRAS NOTCH2NLC KRTAP59 FBN1 KRTAP103 FHL5 ADAMTSL4 NOTCH2NLA	B2283 B2285 B2280 B2275 B2290 B2288 B2434
G299	ADAMTS-13	F8 VWF	B1191 B1180 B1206 B1159 B1190 B1204 B1181 B1197 B1199 B1900 B1897
G315	ADAMTS-3	CCN2	B1424
G324	ADAMTS-2	ALB HPX DCN CTSB DKK3	B1621 B1640 B1623 B1618 B1627 B1620 B1629
G326	ADAMTS-2	A2M	B1641 B1638 B510 B1626 B1643
G328	ADAMTS-2	CST3 COL6A3 PRELP	B1644 B563 B1636 B1631 B1632 B1835
G334	ADAMTS-14	A2M ALB HPX DCN CTSB VCAN DKK3	B1485 B1499 B1486 B1497 B1493 B1410 B1496 B1481 B1498 B1472
G336	ADAMTS-14	CST3 COL6A3	B1480
G340	ADAMTS-14	PRELP	B1474
**G341**	ADAMTS-2, -3, -14	COL5A1 COL1A2 TLL1 F13A1 CFB C3 KNG1 IGHG3 COL1A1 COL2A1 COL3A1 COL4A1 APOA1 APOA2 APOH FN1 AHSG GC ANXA1 COL5A2 KLK1 ANXA2 MMP3 CFH VIM LGALS1 CTSH C4B COL11A1 COL6A1 COL6A2 BMP1 ANXA8 COL11A2 MIF TCN2 OGN BGN MPZ CTSS COL5A3 GRN HLAA SERPINB6 LGALS7 LUM ACTA2 HBB CAV1 TGFBR3 COL14A1 DPT TMED1 ITIH4 POSTN PDIA6 PCOLCE CDSN COL24A1 SUSD4 DMKN CAVIN1 AEBP2 COL27A1 UNC80 CPA4	B1404 B1340 B1378 B1350 B1391 B1360 B1380 B1492 B1407 B1415 B1399 B1341 B1394 B1362 B1349 B1393 B1403 B1374 B1483 B1390 B1637 B1409

**Fig 7:**
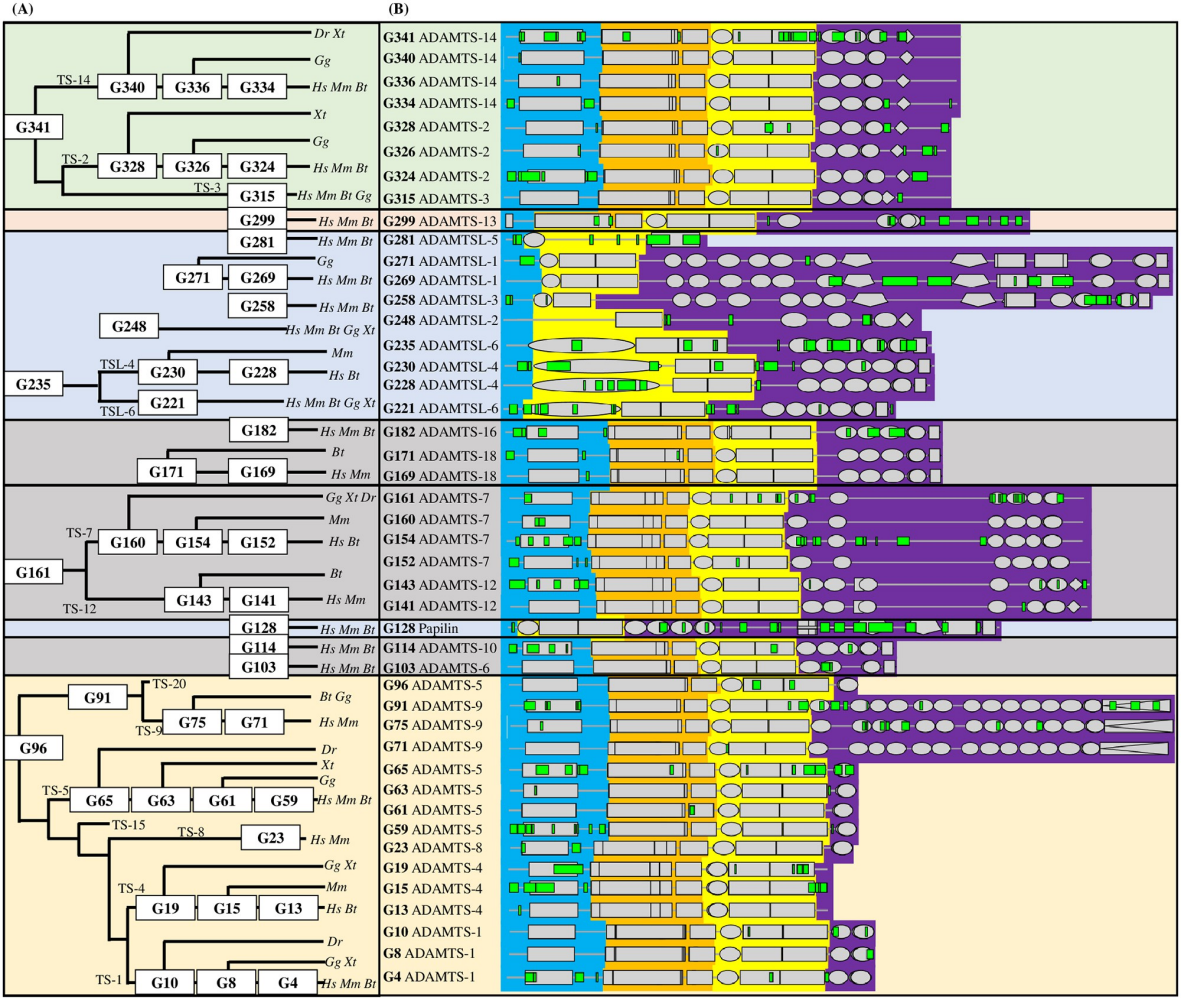
Location in *H. sapiens* paralogs of the modules involved in the 45 events of module(s)-PPI(s) co-appearance. (A) ADAMTS-TSL phylogeny indicating the 45 ancestral nodes (labels in white boxes) corresponding to the 45 module(s)-PPI(s) co-appearance events. TS, ADAMTS; TSL, ADAMTSL; *Hs*, *Homo sapiens*; *Dr*, *Danio rerio*; *Xt*, *Xenopus Tropicalis*; *Gg*, *Gallus gallus*; *Mm*, *Mus musculus*; *Bt*, *Boss Taurus*. (B) Each line corresponds to a H. sapiens protein chosen as representative of the ancestors, and onto which the ancestral module signature is reported. The Pfam domains are represented as grey boxes and the gained modules as green marks. Each sequence is divided into 3 regions; 1) the N-terminal region that contain the propetide in ADAMTS (**blue**), 2) the central region including the catalytic domain and the disintegrin domain (**orange**) and the central TSP1, the cys-rich domain and the spacer (**yellow**) and 3) the variable ancillary region from the end of the spacer to the C-terminal end (**purple**).

Next, we looked at the module location of the 45 signatures along the human protein sequences descending from the corresponding ancestor genes (Fig 7). Of note, all the modules of the signatures are not necessarily found in human proteins, because some could have been subsequently lost or could have diverged in human. Over the 355 modules involved in the 45 signatures, 283 were present in the human descendants. Based on this view of the module signatures associated with PPI co-occurrence events, we observed a wide diversity in the distribution of modules that were gained either within, overlapping with, or outside the known classical domains. Moreover we showed that the variable ancillary region (colored in purple in Fig 7) contained the highest number of modules (49%, 139 among 283). This observation was consistent with the variability of the ancillary region and its known role in mediating specific interactions of ADAMTS-TSL [1]. Note that 30% (86 among 283) of the modules were present in the N-terminal region (colored in blue in Fig 7) which mainly corresponds to the propeptide, a domain known to evolve more rapidly than proper domains thus allowing specialization of function [42]. Importantly, the central region which includes the metalloprotease and disintegrin domains in ADAMTS (colored in orange and yellow in Fig 7) showed few modules 20% (58 among 283). All the variability of the central region was concentrated in the spacer that contained 26 of the 58 modules. Overall, our data supported evidence for the gain of new modules of conserved sequences, possibly associated to a gain of function in the N-terminal, C-terminal, and spacer regions of ADAMTS-TSL, which are regions known to be involved in the emergence of new functions (see our Discussion section on propeptides). These 45 module-PPI associations deserved further investigation, and we searched the literature for experimental evidence supporting putative roles in PPIs for some of our candidate modules.

To further explore the ability of our model to predict associations between conserved sequence modules and PPIs, we focused on two specific cases: first, ADAMTS-7/ADAMTS-12 and their common substrates CCN2 and COMP and, second, the hyalectanase group. In both cases, we compared the model predictions with experimental data to validate the method as well as to propose new insights into the underlying evolutionary processes. The CCN2/COMP PPI case highlights the convergent evolution of PPIs during ADAMTS-TSL evolution while the hyalectanases case explores the sequence evolution of an entire gene subgroup up to ADAMTS-5, a member of this subgroup that shows significant interaction specificity.

### Convergent evolution of COMP and CCN2 interactions with ADAMTS

**Fig 8:**
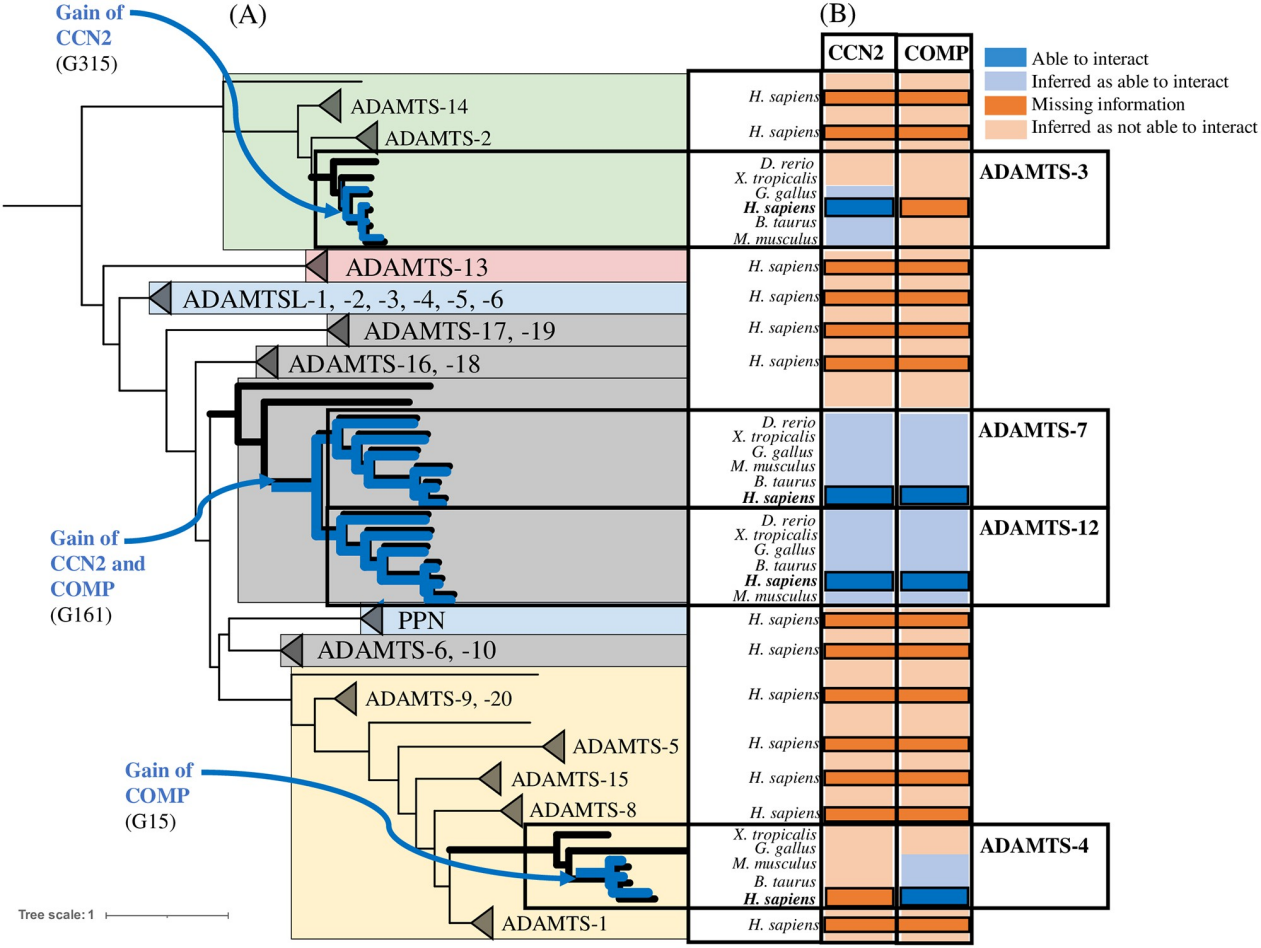
Convergent evolution of COMP and CCN2 interactions with ADAMTS-TSL. COMP and CCN2 interactions with ADAMTS-TSL are associated with independent module signatures acquired during evolution. (A) Phylogenetic tree of ADAMTSs with magnification of phylogenetic ADAMTS subtrees involved in COMP and CCN2 PPIs. (B) Heatmap: the published interactions of *H. sapiens* ADAMTS-3, ADAMTS-4, ADAMTS-7 and ADAMTS-12 with COMP and CCN2 are represented as dark blue boxes. The gains of the PPIs (inferred by PastML) are represented by the internal nodes: G315 (the ADAMTS-3 Amniota ancestor), G161 (the ADAMTS-7 and ADAMTS-12 paralogs ancestor) and G15 (the ADAMTS-4 mammalian ancestor) for CCN2, CCN2/COMP and COMP respectively. The PPIs inferred by PastML are represented as light blue boxes. At the opposite the absence of interaction (missing information) between ADAMTS and CCN2 and COMP is represented as dark orange boxes (for human proteins) and the absence of interaction inferred by PastML is represented as light orange boxes (for non human proteins).

Among the 26 *H. sapiens* ADAMTS-TSL proteins, the literature curation (step 4) identified ADAMTS-3, ADAMTS-4, ADAMTS-7 and ADAMTS-12 as interacting with the cartilage oligomeric matrix protein COMP and/or the connective tissue growth factor (CTGF alias CCN2). The role of ADAMTS-7 and ADAMTS-12 in the pathogenesis of arthritis was firstly supported by their ability to cleave COMP [43, 44]. More recently, CCN2 was also identified as a substrate of ADAMTS-7 and ADAMTS-12 [45, 46]. Since the structures of ADAMTS-7 and ADAMTS-12 are closely related, they form their own subgroup and it is not surprising that they share common substrates [47]. However, the identification of CCN2 and COMP as substrates of the distant ADAMTS, ADAMTS-3 [35] and ADAMTS-4 [48], respectively is less expected. As shown in Fig 8 and consistent with the PPIs inference mediated by PastML, the interaction with CCN2 or COMP most likely occurred twice during the evolution of ADAMTS-TSL. On the one hand, both PPIs (CCN2 and COMP) appeared in G161, the last common ancestral gene of ADAMTS-7 and ADAMTS-12. On the other hand, the COMP PPI was gained at G15, the last common ancestral gene of *H. sapiens, B. taurus, M. musculus* ADAMTS-4 and the CCN2 PPI was gained at G315, the last common ancestral gene of the *H. sapiens, B. taurus, M. musculus, G. gallus* ADAMTS-3.

**Fig 9:**
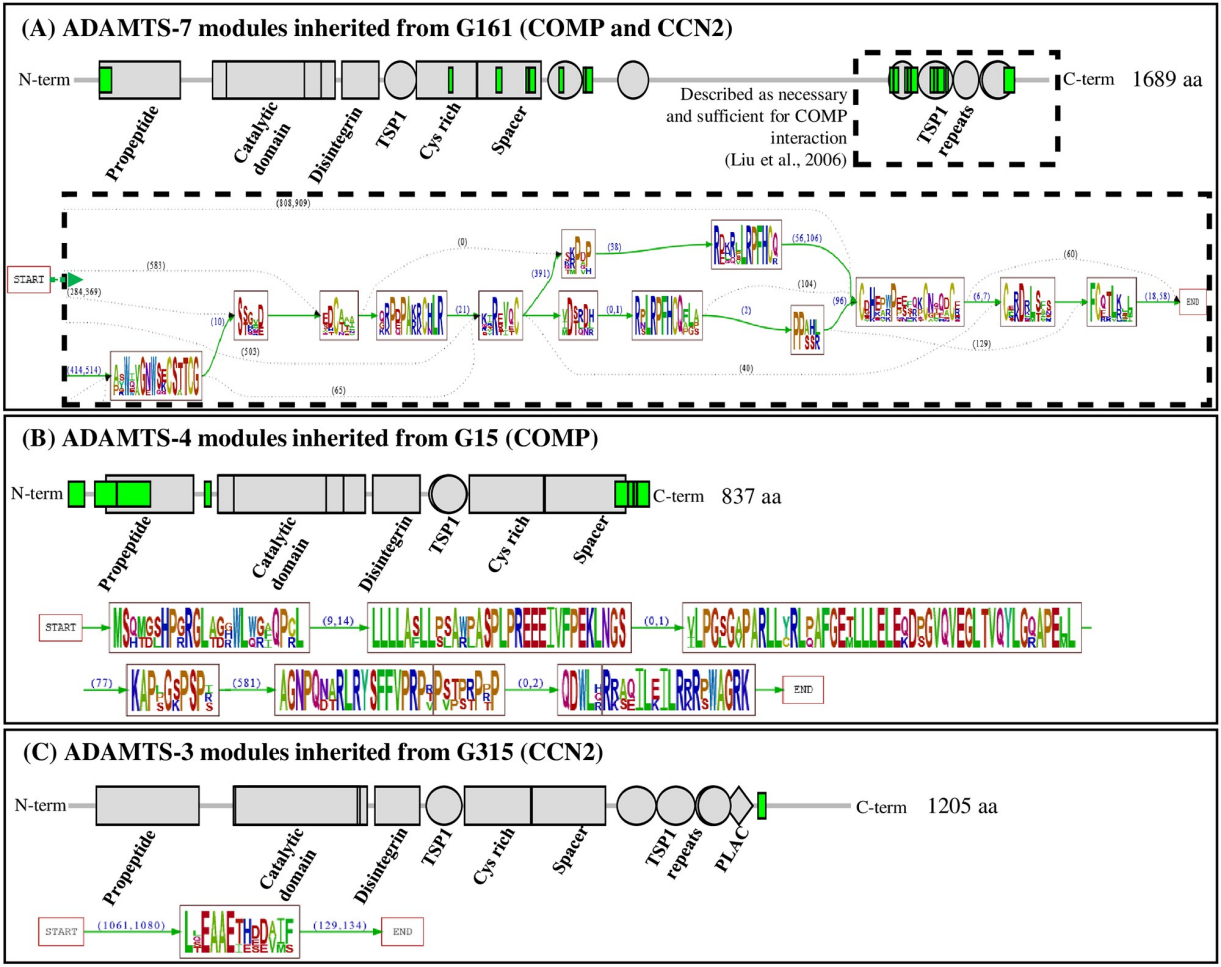
Three module signatures are associated with COMP and/or CCN2 PPIs. The ancestral module signatures reported on the descendant proteins in *H. sapiens*: (A) ADAMTS-7, (B) ADAMTS-4 and (C) ADAMTS-3. The location of the modules is represented as green boxes along with the location of the Pfam domain represented as grey boxes (top panel) while the content of the modules is represented by raw sequence logos [49] using the Protomata visualization [14], which displays also the chaining of the modules with arrows labeled by the minimal and maximal distances between the modules in the sequences of descendants (bottom panels). Because of the size of the G161 module signature, only the modules in the region of interaction with COMP are shown in (A).

To characterize the PPI changes associated with the ancestral genes G161, G15 and G315, we focused on the modules predicted to appear in those ancestors. We first investigated the module signature at G161 and we observed that the 25 modules gained were primarily localized in the TSP1-repeat of the ancillary region (Fig 9A). Importantly the four C-terminal TSP1 motifs corresponded to a region previously shown to be sufficient and necessary for the interaction of ADAMTS-7 and ADAMTS-12 with COMP [43, 44] and CCN2 [45, 46]. However, COMP was also identified as a substrate of ADAMTS-4 that lacks the 4 C-terminal TSP1 motifs suggesting that the interaction with COMP was associated with different sequences. Consistent with this hypothesis, we observed 8 gained modules at G15, the Mammalian ancestor of ADAMTS-4 (Fig 9B), that were localized at both the N-terminal (including peptide signal and part of the propeptide) and the C-terminal parts of the sequence. In addition, no module of the G15 signature was shared with the G161 signature. Similarly, we observed a specific gained module at G315, the Amniota ancestor of ADAMTS-3 (Fig 9C) that localized in the end of the ancillary region and this unique module was not found in the G161 signature. These analyses revealed that, despite similar localization in the ancillary region, the three module signatures (G161, G15, G315) were completely different in both their module sequences and their domain contexts. Overall, our data support the hypothesis that the interactions with COMP and CCN2 were each acquired independently at two distinct time points during ADAMTS-TSL evolution. These two cases of convergent evolution allowed us to propose 3 distinct module signatures, each characterized by specific modules in the C-terminal part of the ancillary region. In these particular cases, when convergent PPI evolution occurred, each independent PPI acquisition could be associated with a different ADAMTS-TSL interaction site.

### The paralog expansion of hyalectanases highlights the functional specificity of ADAMTS-5

**Fig 10:**
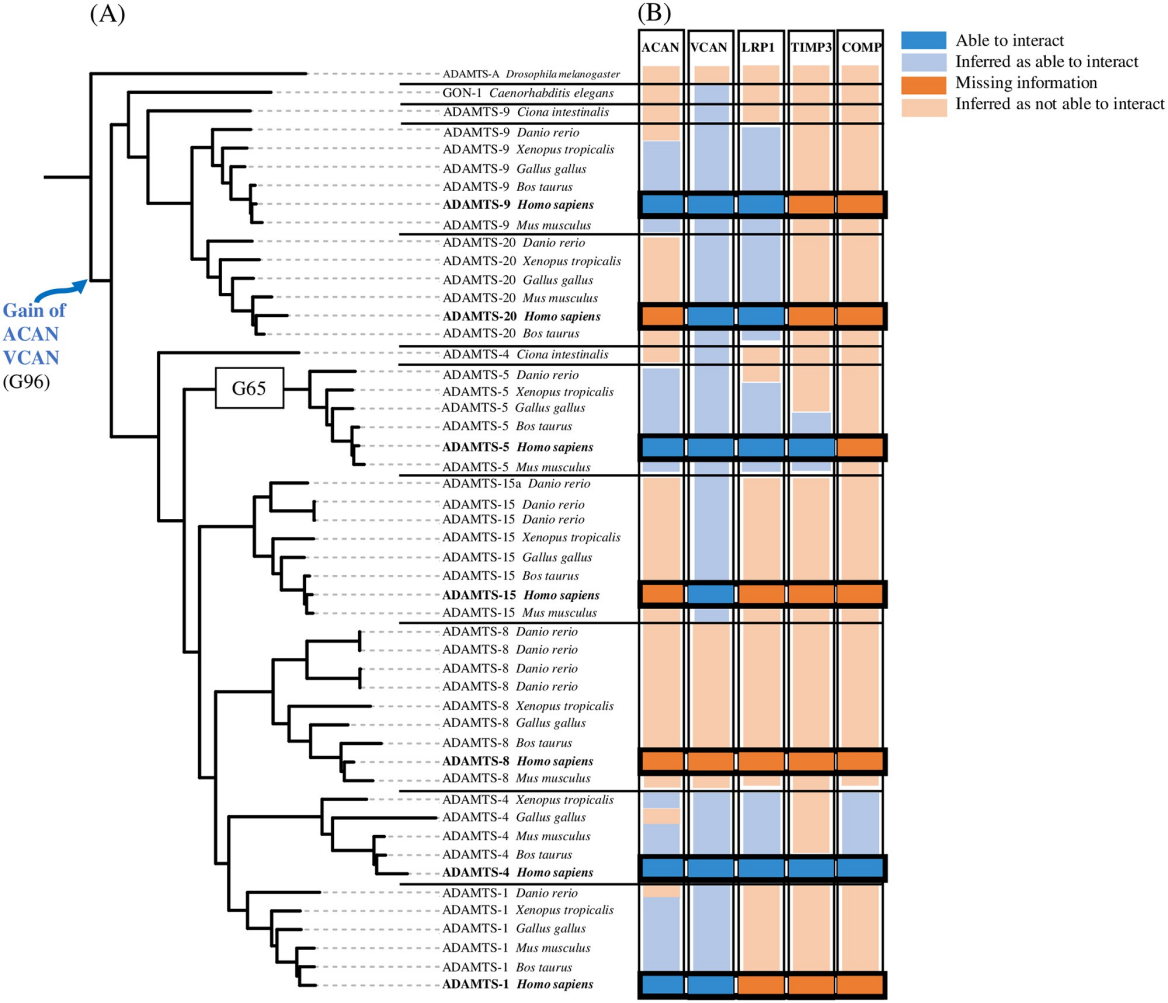
Evolutionary histories of hyalectanases PPIs. (A) Hyalectanase tree. The last common ancestor of the human hyalectanases is the G96 gene node. The gain of the ACAN and VCAN PPIs was inferred at the G96 gene node. (B) Heatmap: the published interactions of *H. sapiens* hyalectanases with ACAN, VCAN, LRP1, TIMP3 and COMP are represented as dark blue boxes. The PPIs inferred by PastML tool are represented as light blue boxes. At the opposite the absence of interaction (missing information) between hyalectanases and ACAN, VCAN, LRP1, TIMP3 and COMP is represented as dark orange boxes (for human proteins) and the absence of interaction inferred by PastML is represented as light orange boxes (for non human proteins).

The hyalectanase family is composed of 7 members (ADAMTS-1, ADAMTS-4, ADAMTS-5, ADAMTS-8, ADAMTS-9, ADAMTS-15 and ADAMTS-20) which degrade proteoglycans such as aggrecan (ACAN), versican (VCAN), brevican and neurocan [50]. The last common ancestor of the hyalectanase genes was identified as the G96 gene for which the phenotype of interaction with ACAN and VCAN was gained (Fig 10A). Note that some hyalectanases shared other interactants such as LRP1 which was identified as partner of ADAMTS-4, ADAMTS-5, ADAMTS-9 and ADAMTS-20 (Fig 10B).

According to the literature, ADAMTS-4 (aggrecanase-1) and ADAMTS-5 (aggrecanase-2) are the main hyalectanases involved in the degradation of aggrecan (ACAN), the major component of articular cartilage. While ADAMTS-1 [51] and ADAMTS-9 [52] interact with aggrecan, they have lesser degrading activity. Although the activity of recombinant ADAMTS-5 was first shown to be twice as slow as that of ADAMTS-4 *in vitro* [53], ADAMTS-5 is the major aggrecanase in physiological conditions [9–11]. The interaction site with aggrecan involves the spacer domain for ADAMTS-4 [54] and both the cystein-rich and spacer domains for ADAMTS-5 [11]. Other substrates of ADAMTS-4 and ADAMTS-5 have been identified and some are common such as VCAN [51, 55] and others are different such as COMP which is only cleaved by ADAMTS-4 and not by ADAMTS-5 [48]. In addition, ADAMTS-4 and ADAMTS-5 share interactions with other proteins such as TIMP3 through their C-terminal domains [56] or such as LRP1 through different domains. Indeed, the cystein-rich and spacer domains of ADAMTS-4 were involved in the interaction with LRP1 whereas the thrombospondin 1 and spacer domains of ADAMTS-5 were involved in the interaction with LRP1 [57]. It is important to note that unlike other hyalectanases, the propeptide of ADAMTS-5 is cleaved extracellularly suggesting specific features [58].

**Fig 11:**
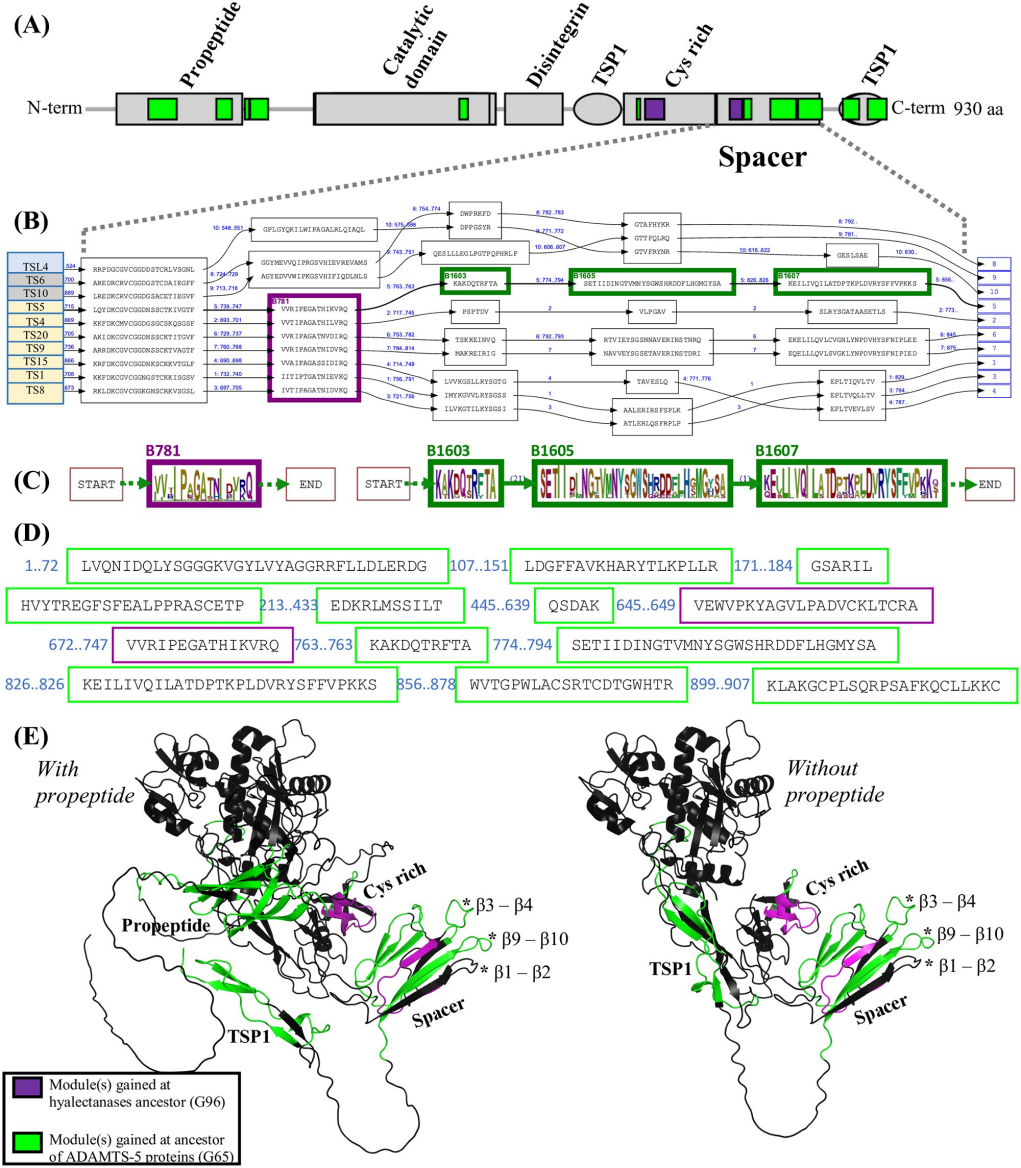
Module signatures of ADAMTS-5 and hyalectanase proteins. (A) Location of G65 and G96 signature modules on *H. sapiens* ADAMTS-5 (NP_008969.2). The protein domains are shown in grey, the modules gained at the ADAMTS-5 ancestral gene G65 are shown in green and the modules gained at the G96 hyalectanase ancestral gene are shown in purple. (B) Excerpt of the PLMA restricted to the sequences of *H. sapiens* hyalectanases and three non hyalectanases (ADAMTS-6, ADAMTS-10 and ADAMTSL-4) in the spacer domain. PLMA blocks defining modules are shown as boxes containing the module segments. The succession of the module segments of each sequence is indicated by arrows labeled by a numbering of the sequences displayed (from 1 to 10 here), completed with the interval of sequence positions skipped if the segments are not contiguous. (C) Sequence logos as in Fig 9 of the modules gained at G96 (left) and G65 (right) in the spacer. (D) All the segments of *H. sapiens* ADAMTS-5 (NP_008969.2) in G65 and G96 signature modules (E) Predicted structures of the *H. sapiens* ADAMTS-5 protein with and without propeptide, colored with G65 and G96 modules. The three hypervariable loops, β1-β2, β3-β4 and β9-β10 previously described in Santamaria et al, 2019 [59] are marked by *.

These experimental observations suggest a functional specificity of ADAMTS-5 compared with ADAMTS-4 and other hyalectanases. Therefore, we examined the module signature in the ancestor of ADAMTS-5 proteins that may account for its uniqueness. At G65, the last common ancestor of all ADAMTS-5 orthologs (Fig 10A), 12 modules were gained, 11 of them being present in ADAMTS-5 *H. sapiens* (green boxes in Fig 11A). These modules are mainly localized in the propeptide domain, the spacer and the C-terminal TSP1, the two other domains being in the catalytic site and the cystein-rich domain. Looking at the ancestor G96 of the hyalectanases, the 2 gained modules are localized in the cystein-rich domain and the N-ter of the spacer domain (purple boxes in Fig 11A).

Because the spacer domain at G65 and G96 genes accumulated many changes, we further analyzed the sequences in this domain which has been shown to be involved in the interaction with aggrecan. The Fig 11B shows all the modules found in the spacer by the PLMA on sequences of the *H. sapiens* hyalectanases as well as three additional non hyalectanase sequences (ADAMTS-6,-10 and ADAMTSL-4) to illustrate the block conservation with regard to close and distant paralogs of hyalectanases. The B781 block (purple box in Fig 11B and 11C) is the specific block of hyalectanases in the spacer region. After this block, the only conservation blocks detected in hyalactanase paralogs localized between ADAMTS-1, ADAMTS-8 and ADAMTS-15 and between ADAMTS-9 and ADAMTS-20. It is important to note that the two close aggrecanases ADAMTS-4 and ADAMTS-5 displayed distinct conservation blocks in this spacer region, ADAMTS-5 being characterized by three specific blocks, B1603, B1605 and B1607 (green boxes in Fig 11B and 11C) shared only with its orthologs. The sequences of B1603 and B1607 blocks overlapped with the hypervariable loops *β*3-*β*4 and *β*9-*β*10 that were previously reported to mediate the specificity of ADAMTS interaction [59]. Note that no block was identified in sequences corresponding to the third hypervariable loop *β*1-*β*2 described by Santamaria et al [59]. The *G*65 specific conservation identified by block B1605 matched instead with the *β*6-*β*7-*β*8 region and the surrounding loops (using beta strand numbering from [59]).

The full set of segments of *H. sapiens* ADAMTS-5 belonging to G65 and G96 module signatures are shown in Fig 11D. We crossed also our inference of G65 and G96 module signatures with the prediction of their spatial localization in the *H. sapiens* ADAMTS-5 protein. AlphaFold 2.1.14 [60] was used in the ColabFold 1.3.0 local environment [61] to predict the 3D structure of *H. sapiens* ADAMTS-5. Since ADAMTS-5 is activated in the extracellular space [58], we explored the structures of ADAMTS-5 with and without the propeptide, the peptide signal being removed from the sequence. The prediction was made from both sequences by launching the command colabfold_batch with the parameters --num-recycle 3 --num-models = 5 --amber. The two resulting first ranked relaxed models colored by the location of G65 and G96 modules are shown in Fig 11E. The predicted structures are available as S1 and S2 Files and the corresponding pLDDT and PAE plots are presented in S1 and S2 Figs. It is important to note that removing the propeptide does not affect the prediction much, the main difference being in the movement of the C-terminal TSP1 domain towards the place left empty by the propeptide. The pLDDT and pTM scores which are respectively 90.3 and 0.79 without the propeptide changed to 78.3 and 0.592 with the propeptide, showing a stronger confidence in the prediction of the model without the propeptide.

Interestingly, although module segments may be distant in the sequence, they are all located in the same area of the predicted 3D structures at the interface of the protein, around a conserved ADAMTS-TSL core consisting of the central TSP1 domain and the C-term of the cystein-rich domain, with the exception of the module segment in the catalytic domain. In particular, the segments of the G65 and G96 signature modules from the cystein rich and spacer domains seem to form a localized sub-unit made of a beta sheet in the spacer facing the conserved segments in the cystein rich domain. The segment of the G65 module in the cystein rich domain is also interestingly predicted in a loop with an orientation similar to the β1-β2, β3-β4 and β9-β10 hypervariable loops identified by Santamaria et al [59] and the *β*6-*β*7-*β*8 loops from the spacer domain. Note that the specific G65 module segments present in the C-terminal TSP1 and the propeptide are co-localized near the central TSP1 domain in the predicted structure suggesting a regulatory role for the propeptide.

Together our data summarized in Fig 11 allow us to propose a scenario for the evolution of the spacer domain leading to a specialization of hyalectanases/ADAMTS-5. According to this scenario, a first conserved module appeared in the hyalectanase ancestor (positions 748–762 in Fig 11D) prior to the specialization of the three specific ADAMTS-5 conservation modules (positions 764–773, 795–825 and 827–855 in Fig 11D). Two of these three specific modules coincided with already identified regions by Santamaria et al [59]. The third one, appearing in an interesting location of the predicted structures, and the absence of conservation in the *β*1-*β*2 region of ADAMTS-5 provided novel insights into the spacer domain. Moreover, these conserved modules in the spacer are likely to be functionally linked to the specific modules in the cystein rich domain from their respective orientation in the predicted 3D structures. Besides, our data highlight also specific conservations predicted to be spatially co-localized in the propeptide and C-term TSP1 regions, which are challenging to interpret and are of interest to guide further studies.

## Discussion

Emerging new combinations of domains plays a major role in the evolution of modular organization of proteins, and the emergence of adaptive traits. This phenomenon has been shown to be predominant in the evolution of proteins involved in the extracellular matrix, an interconnected network composed of hundreds of macromolecules that includes the multidomain protein family ADAMTS-TSL [62–66]. Here we investigated the ADAMTS-TSL family in order to identify functional sequence regions in their modular structure based on two widely used methods for functional prediction [67], namely sequence conservation analysis [68] and phylogenomic inference [15]. We provided an extension of phylogenomics inference to modules using Domain-Gene-Species (DGS) reconciliation [24]. This task is challenging because of the large paralogous expansion in that family (26 paralogs in human) and the large number and diversity of annotated protein domains (from 5 to 9 in human paralogs).

### Inference of the ADAMTS-TSL phylogeny

The evolutionary history of the ADAMTS-TSL protein family has previously been studied [69–72]. Using ADAM proteins as the outgroup, the present work provided a rooted phylogeny of 173 ADAMTS and ADAMTSL from 9 bilateria species that was consistent with the main conclusions of these previous studies, and especially with a family expansion in vertebrates, possibly driven through whole genome duplication events [72]. Note that similarly to the rooting using one Porifera ADAMTS sequence [72], our rooted phylogeny identified the procollagen peptidases as the first to diverge within the ADAMTS and ADAMTSL. Importantly and unlike other studies, we used both a reconciliation method that identified paralogous gene duplication and speciation events, and a correction method taking into account the species tree, that corrected stochastic errors in the gene tree. As previously described by Brunet et al. [72], we identified a monophyly of the procollagen peptidases (ADAMTS-2, -3 and -14) and a monophyly of the hyalectanases (ADAMTS-1, -4, -5, -8, -9, -15 and -20) with consistent relations within these two clades. In adition, we also identified as monophyletic the COMP proteases (ADAMTS-7 and ADAMTS-12) and the other pairs of enzymes known as closely related paralogs (i.e. ADAMTS-6 and ADAMTS-10, ADAMTS-16 and ADAMTS-18, ADAMTS-17 and ADAMTS-19). Importantly these groups are found robust to the choice of the considered sequence sets, as shown by gene re-sampling assays (See S1 Appendix).

An interesting new finding is the monophyly of the ADAMTSL group while papilin, a member of the ADAMTSL protein family, clustered in a distinct group related to ADAMTS-6 and ADAMTS-10. Note that these two distinct groups were not supported in all the gene re-sampling assays (See S1 Appendix). However our results were in agreement with an origin of ADAMTSL and papilin within the ADAMTS and a loss of the catalytic domain [73–75], even if it remains unclear whether this event has happened once or more.

### Sequence decomposition into modules and evolution of module composition

Many studies have used domain composition as a proxy for protein function, as they represent independent structural and functional units (see Cromar et al 2014 [62], for such a study focusing on ECM proteins). Instead, in this work we focused on highly conserved protein modules as a proxy for functional prediction. High local conservation reveals regions that likely confer functional features conserved since a common ancestor [67, 68]. The rational for this assumption is that negative natural selection applying to maintain an important phenotype (including functions) therefore applies at the genotype level, avoiding sequence divergence.

Sequence conservation is generally identified using global alignments, protein structures or features and motifs identified *a priori* [76]. Few alignment methods allow the identification of locally conserved sequences without *a priori*. Here, we used a new implementation of the alignment program Paloma from the Protomata suite (See S2 Appendix). Segments within the same module are strongly similar, have the same size and can only differ through substitutions. Thanks to this method, we identified modules representing highly conserved segments shared by some of, and not necessarily all, the protein sequences. By applying Domain-Gene-Species (DGS) reconciliation to module trees, we next identified the gene ancestors which acquired new modules. This provides a phylogenetic characterization of the module. Proteins derived from the ancestral gene tend to possess this module, but not the more distantly related proteins.

The modules are gapless: the strongly conserved segments have no visible traces of insertion or deletion events. Hence, a conserved region with insertions and deletions will be modeled by several modules. The number of modules will increase as the number of insertion and deletion augments, and the size of the modules will decrease, leading to a more fragmented characterization of the region focusing on the conserved parts only.

The value of the conservation threshold parameter can be particularly decisive for the results. Actually, relaxing this threshold, it might be possible to get longer modules involving more genes and older common ancestors. Thus a module gain or loss does not necessarily account for the ancestral locus gain or loss, but indicates that significant differences exist between subsets of segments originating from that locus. For instance, the gain of a conserved module in a given lineage would correspond to a sequence divergence above the chosen conservation threshold. In such a scenario, two different modules may correspond to homologous sequences that diverged from an older ancestral locus. In this study, the modules were identified on the basis of a highly conservative diagonal’s similarity threshold. Let us emphasize that our module decomposition identified segments conserved in all the ADAMTS and belonging, as expected, to shared domains such as the catalytic domain, suggesting that the approach and the chosen threshold are adapted to our study case.

Our innovative approach of combining partial local multiple alignment with the DGS reconciliation method allowed us to define module signatures (i.e., modules acquired simultaneously during gene evolution). A module signature thus consists of one or several independent modules, so that one of their distinguishing feature is the possibility of being discontinuous along the sequences.

### Convergent Protein-Protein Interactions to increase ECM complexity

Protein-Protein Interactions play fundamental role in the structure of the ECM [62, 66] and are key features to understand the functions of ADAMTS-TSL as demonstrated for the formation of microfibril and its deregulation in Acromelic dysplasias [4, 77]. Here, we reconstructed 278 ancestral PPIs along the ADAMTS-TSL phylogeny and we identified the convergent appearance of ADAMTS interactions with COMP and CCN2. Because the interaction with COMP involves four TSP1 repeats in ADAMTS-7 and ADAMTS-12 [43, 44] that are not present in the ADAMTS-4, interaction sites are different. We propose that such convergences are favored by selective pressures leading to the increase of the ECM complexity by increasing the possible interactions among ECM proteins. Importantly, ADAMTS4, ADAMTS7, and ADAMTS12 degrade the COMP protein that must interact with the catalytic site in the metalloprotease domain. However, the enzyme-substrate interaction is transient in nature and requires the substrate to be “fixed” in the vicinity of the catalytic site. This local organization depends on the molecular networks where the enzyme-substrate complex takes place and could explain the involvement of different module sequences according to ADAMTS. Cromar et al (2014) [62] demonstrated that novel domain arrangements have contributed to increased ECM complexity in vertebrates, multiple possible domain combinations contributing to the emergence of new functions. Similarly, increasing the possible ADAMTS-TSL interactions may contribute to increase ECM complexity. Hence, whereas domain combination, appearance and shuffling received attention in understanding the functions of proteins involved in the ECM [64], our hypothesis suggests that convergent interactions between ECM proteins, such as observed here for the ADAMTS, might constitute another mechanism to diversify the functions of ECM proteins.

### Ancestral module signatures as putative functional motifs

The identification of functional sites in a protein is a prerequisite to understand its molecular action and to develop efficient therapeutic approaches [76]. Numerous computational prediction methods have been developed [78] providing a large diversity of tools for biologists. Among them, methods based on conserved sequence analysis are widely used. As mentioned above they are relying on the principle that proteins inherit their phenotypes and genotypes from a common ancestor. However having similar domains in common does not necessarily indicate that proteins have similar functions especially in the case of multidomain proteins such as ADAMTS-TSL. Consistent with this is the failure to target the activities of MMPs via their catalytic domain [79]. By combining PPI annotations and sequence conservation analysis, we developed an original approach that predicts module signatures composed of non linear sub-sequences sets associated with the function of Protein-Protein Interactions. Note here that any phenotype of interest might be associated with the ancestral module signature. In the present work, we chose PPIs as the phenotype of interest and we identified 45 module signatures as putative functional motifs.

Our method is supported by the identification of module signatures that matched functional motifs previously identified, such as PPIs involving COMP and ADAMTS-7/ADAMTS-12 [43, 44], or PPIs involving ACAN and the spacer domain of aggrecanases [80]. In addition the module signatures of the hyalectanases revealed differences that might explain the differences of activity against the substrate ACAN [9–12, 51, 52, 52]. The physiological aggrecanase ADAMTS-5 showed a unique signature containing 12 modules spread over the sequence but spatially very close in the structure of the protein, thereby defining a putative specific area of interaction with ACAN. Importantly two hypervariable loops of ADAMTS-5, shown to be important for its aggrecanase activity, overlapped with our predicted signature supporting its reliability [81]. Although monoclonal antibodies and small molecules have been developed to inhibit the deleterious effect of ADAMTS-5 in osteoarthritis [82], side effects and lack of specificity have prevented their progression towards clinical trials. In this context, our specific sequence signature could be valuable for the design of novel inhibitory molecules.

### The propeptide of ADAMTS as a source of divergence

Like other ADAMTSs, ADAMTS-5 shows high variability in the propeptide domain (Fig 7) that has been previously described as an evolutionary module and transient protein domain [42]. Propeptides evolve more rapidly than other domains allowing diversification of gene functions. Our observations are consistent with the specificity of these propeptides while suggesting module signatures specific to functional dissimilarities within the ADAMTS-TSL family. The appearance of modules in the N-terminal part of ADAMTS (signal peptide and propeptide) is intriguing since the signal peptide and the amino-propeptide are usually processed for protease secretion and activation thus acting as sorting signal [83] and inhibitory [84] peptides, respectively. However, other functions have been attributed to propeptides including chaperone-assisted intramolecular protein folding, protein sorting, and mediation of precursor interaction with its partners [42, 84]. Most ADAMTS contain a convertase cleavage site, however processing of ADAMTSs affects the localization and activity of the protein differently. For ADAMTS9, the propeptide acts as both an intramolecular chaperone and a sorting protein to target ADAMTS9 to the membrane where the protease is then processed by furin [85, 86]. Unlike most ADAMTSs, furin processing of ADAMTS9 leads to an inactive enzyme. Similar to ADAMTS9, the propeptide of ADAMTS7 acts as a chaperone and ADAMTS17 undergoes autocatalytic processing independent of propeptide cleavage [87]. ADAMTS13 is also characterized by propeptide independent regulation. The functional specificity of ADAMTS13 may be related to its very short propeptide that does not affect protease activity against Von Willebrand factor [88]. Consistent with the heterogeneity of propeptide functions in ADAMTS, Kutz et al [89] demonstrate that the propeptide of ADAMTS10 is processed by convertases with very low efficiency and remains associated with the catalytically active protein. The associated propeptide may induce a conformational change that affect the interaction with fibrillin 1, contributing to microfibril biogenesis rather than in fibrillin turnover. In contrast, its closest homolog, ADAMTS6, showed a functional furin consensus sequence [90] and no interaction with fibrillin or contribution to microfibril biogenesis has been demonstrated to date [91]. Specific regulation was also demonstrated for ADAMTS5 which is characterized by a unique extracellular processing [92]. While processing of ADAMTS5 is required for its activity against versican and aggrecan, cleavage of the propeptide is mediated by extracellular proprotein convertases, suggesting that the propeptide contributes to adequate localization in the extracellular matrix where processing takes place. This specific mechanism may be associated to the high efficiency of ADAMTS5 activity compared to all other hyalectanases. All of these observations highlight the diversity in the regulation of ADAMTS activity by the N-terminal peptide and are consistent with the known variability of propeptides with respect to the conserved catalytic domain making propeptides “evolutionary specific factors” [42]. This suggests that future development of inhibitory molecules could take into account the specificity of propeptides that regulate ADAMTS activity.

## Conclusion

We propose here a new approach bringing together advanced sequence analysis and phylogenetic methods for identifying conserved sequence modules associated with protein function. This approach is based on an original integration of the evolutionary histories of species, genes, modules and on the prediction of modules and phenotype co-appearances to reveal sets of (non-contiguous) conserved sequence regions defining phenotype specific signatures. We applied this strategy to search for conserved modules in the ADAMTS-TSL family using Protein-Protein Interaction data as phenotypes. Based on a set of experimentally supported ADAMTS-TSL protein interactions we provide novel insight into the evolution and specialization of ADAMTS-TSL interactions. Our framework is generalizable to other protein families as well as to other phenotypes, giving new perspectives to the functional characterization of other multi-functional, multi-domain protein families.

## Supporting information

S1 Appendix Uncertainty analysis of the ADAMTS-TSL phylogenetic tree. (PDF)

S2 Appendix Partial local multiple alignment with Paloma-D. (PDF)

S3 Appendix Browsing the ADAMTS-TSL Itol tree. Itol tree screenshots and usage examples.
(PDF)

S1 Table Description of the species, identifiers and assemblies. (CSV)

S2 Table Quantification of amino acids in longest isoforms compared to splice graph. The whole isoform sequences were examined as splicing graphs and the unconsidered information for all isoforms was quantified.
(CSV)

S3 Table The 214 representative protein sequences dataset: Sequence RefSeq ids and species taxid. (CSV)

S4 Table List of PPIs identified by PSICQUIC analyses. (XLSX)

S5 Table Distribution of PPIs identified by PSCICQUIC analyses according to species. (XLSX)

S6 Table List of ADAMTS substrates identified by manual literature curation. (XLSX)

S7 Table List of Protein-Protein Interactions for ADAMTS-TSL. (CSV)

S8 Table Module composition of the 26 *H. sapiens* ADAMTS-TSL. (CSV)

S9 Table List of the 1059 conserved sequence modules. (CSV)

S1 Fig pLDDT and PAE scores of the ADAMTS-5 AlphaFold’s predicted structure without peptide signal and with propeptide. (PDF)

S2 Fig pLDDT and PAE scores of the ADAMTS-5 AlphaFold’s predicted structure without both peptide signal and propeptide. (PDF)

S1 File The ADAMTS-5 AlphaFold’s predicted structure without peptide signal and with propeptide. (PDB)

S2 File The ADAMTS-5 AlphaFold’s predicted structure without both peptide signal and propeptide. (PDB)
