# Supplementary material for: Phylogenetic inference of the emergence of sequence modules and protein-protein interactions in the ADAMTS-TSL family
Source: PLoS Comput Biol. 2023 Aug 31;19(8):e1011404. doi: 10.1371/journal.pcbi.1011404 (PMC10499240; doi:10.1371/journal.pcbi.1011404)
Supplement: S1 Appendix — (PDF) [file pcbi.1011404.s001.pdf]

Phylogenetic inference of the emergence of sequence modules and protein-protein interactions in the ADAMTS-TSL family

S1 Appendix: **Analysis of the uncertainty in the ADAMTS-TSL phylogenetic tree**

Olivier Dennler[1,2], François Coste[1], Samuel Blanquart[1], Catherine Belleannée[1], Nathalie Théret[1,2*]

1 Univ Rennes, Inria, CNRS, IRISA, UMR 6074, Rennes, France
2 Univ Rennes, Inserm, EHESP, Irset, UMR S1085, Rennes, France

* nathalie.theret@inserm.fr

As ADAMTS-TSL proteins are composed of multiple domains, with many motif and domain repeats, we are cautious about the inferred phylogeny (i.e., the reference tree, Fig 4 in main).

1 The reference gene tree (reminder)

As described in the Materials and Methods, the gene phylogeny was inferred from the 214 representative protein sequences, each being encoded by a single gene (173 ADAMTS and ADAMTSL as the in-group, 41 ADAM as the out-group). This calculation required several steps and resulted in the final phylogeny hereafter referred to as the *reference gene tree*. As a first step, we used the multiple sequence aligner PASTA [1] (default settings) to compute the multiple alignment of the 214 proteins and the maximum likelihood phylogeny inference software RAxML [2] (default settings) to infer a first initial phylogeny. This initial tree was corrected using Treefix [3] taking into account the species tree as defined in the NCBI Taxonomy, thus possibly modifying the topology of the initial tree. The branch lengths (lost by Treefix) were then recomputed using PhyML [4] and the resulting tree was rooted using ADAM proteins as the out-group, resulting in the final phylogeny, designated as the *reference gene tree* (Fig 4 in main).

2 Uncertainty in the ADAMTS-TSL phylogenetic tree

To analyse the uncertainty resulting from the taxon and gene sampling, and to identify robust bi-partitions in our *reference gene tree*, we analyzed different ADAMTS-TSL protein samples. Our study included two types of re-sampling: protein sequence from the **out-group** (i.e., ADAM re-sampling) and **in-group** (i.e., ADAMTS-TSL re-sampling) of the reference gene tree.

We find that internal nodes are robust to both in-group and out-group resampling, whereas recent nodes, notably the subgroup bi-partitions (e.g., ancestor of procollagenase, ancestor of aggrecanase), are relatively robust to both resampling. In other words, the most ancient gene nodes that are not associated with co-appearances are not robust, while the more recent gene nodes are robust. This paper presents co-appearances of fairly recent gene nodes that correspond to robust bi-partitions.

All methods and the resulting trees are presented in the following sections. Sequences were aligned using the PASTA software with default parameters and the phylogeny was inferred using the RAxML software with default parameters. The trees were visualized using the Interactive Tree Of Life software, Itol [5].

3 The out-group gene re-sampling

The out-group gene re-sampling used subsets of the out-group ADAM proteins. We constructed 6 different data-sets, each including all the 125 ADAMTS and 48 ADAMTSL sequences plus a subset of the 41 ADAM out-group sequences including either i) ADAM9 (tree in Fig 1), ii) ADAM12 (tree in Fig 2), iii) ADAM17 (tree in Fig 3), iv) ADAM10 (tree in Fig 4), v) ADAM9 plus ADAM12 (tree in Fig 5) and vi) ADAM17 plus ADAM10 (tree in Fig 6). Then, we constructed the multiple sequence alignment (MSA) using PASTA software [1] and inferred the phylogeny of each data-set using the RAxML software [2]. ADAM out-groups were used to root the trees. Finally we computed the "support values" of the reference gene bipartitions, indicating, for each bipartition, the frequency for the resampled trees among 6 that have the bipartition (Fig 7).

4 The in-group gene re-sampling

The in-group gene re-sampling used ADAMTS-TSL proteins from different species. We constructed an alternative ADAMTS-TSL data-set, composed of 255 known sequences (canonical Uniprot) from six species not considered in our reference data-set (*Rattus norvegicus, Xenopus laevis, Canis lupus familiaris, Felis catus, Anolis carolinensis, Pan troglodytes*). Next, we constructed 20 different data-sets, each containing all 173 ADAMTS-TSL sequences from the reference data-set plus 10 sequences randomly chosen from the alternative data-set. Then, we constructed the multiple sequence alignment (MSA) using PASTA software [1] and inferred the phylogeny of each data-set using the RAxML software [2]. Finally we computed the "support values" of the reference gene tree bipartitions, using these 20 re-sampled trees pruned from the added sequences (Fig 8).

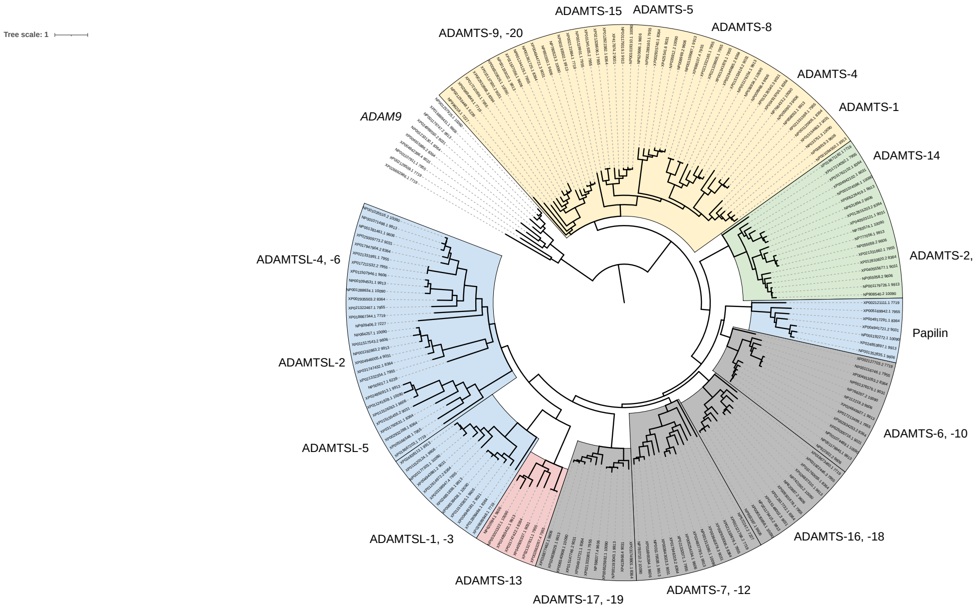

Fig 1. Gene tree with ADAM9 as the out-group 125 ADAMTS, 48 ADAMTSL and 10 ADAM9 sequences were used.

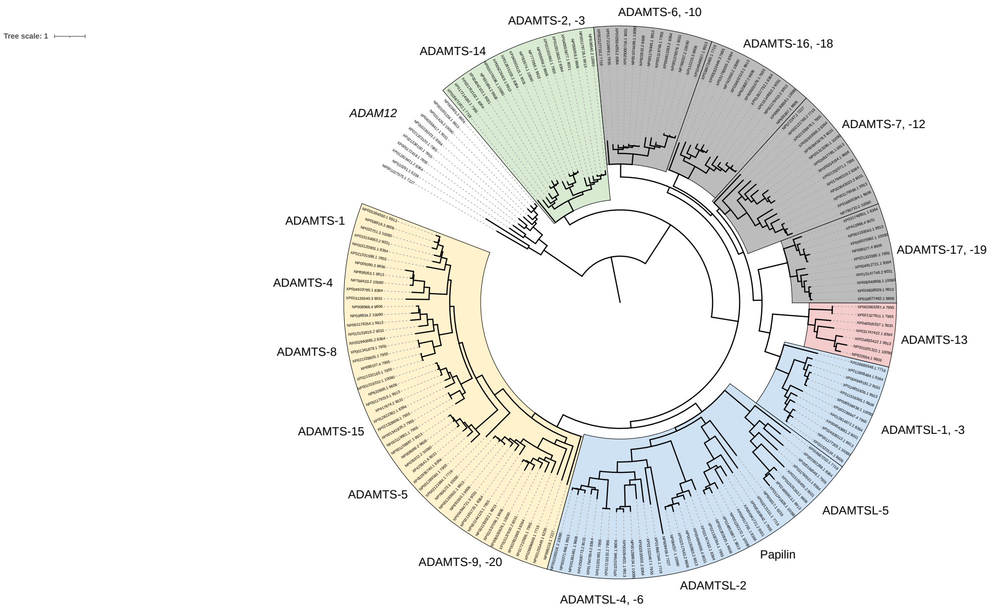

Fig 2. Gene tree with ADAM12 as the out-group 125 ADAMTS, 48 ADAMTSL and 11 ADAM12 sequences were used.

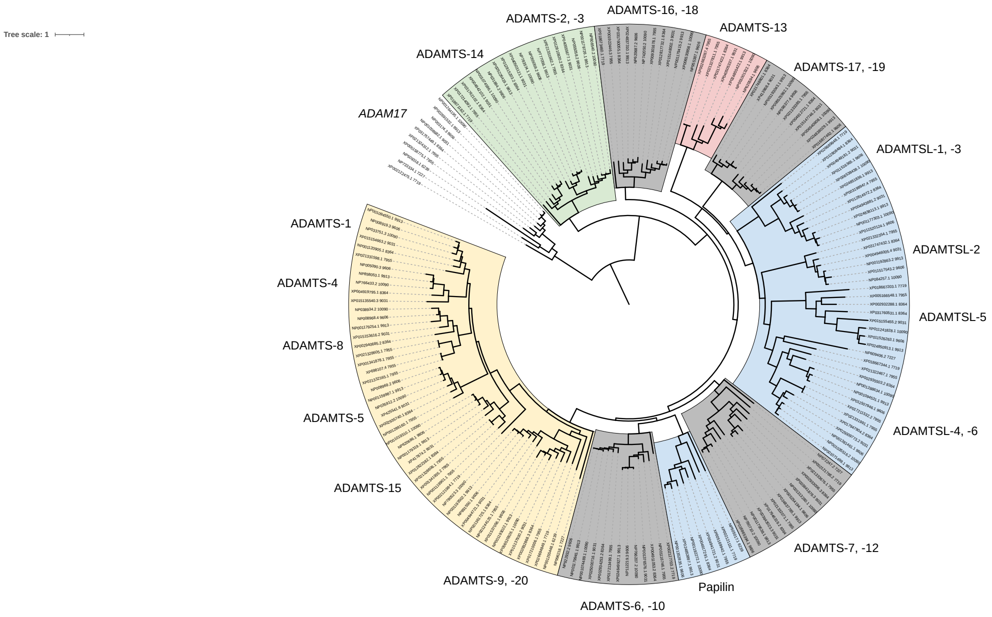

Fig 3. Gene tree with ADAM17 as the out-group 125 ADAMTS, 48 ADAMTSL and 10 ADAM17 sequences were used.

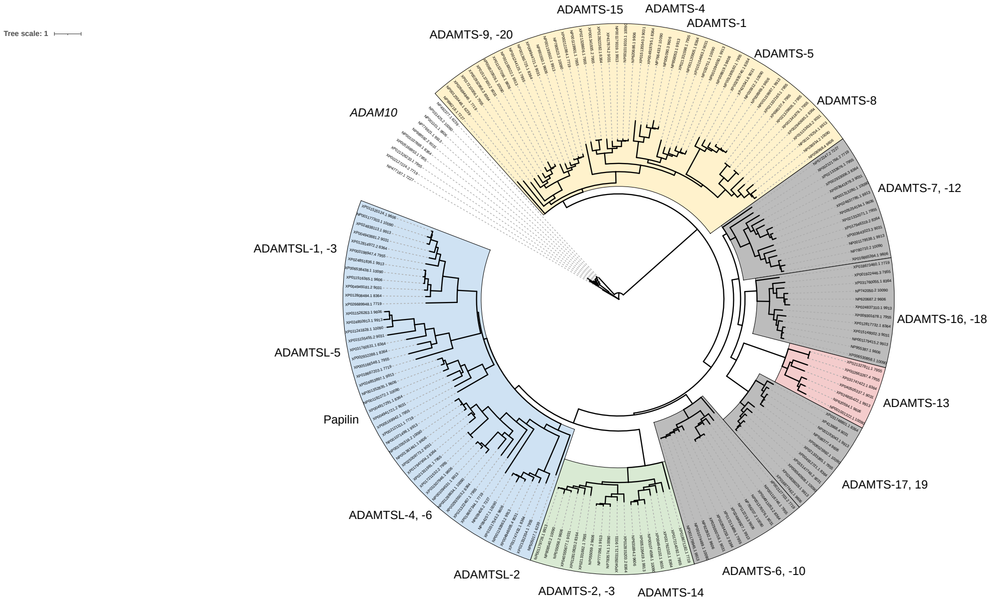

Fig 4. Gene tree with ADAM10 as the out-group 125 ADAMTS, 48 ADAMTSL and 10 ADAM10 sequences were used.

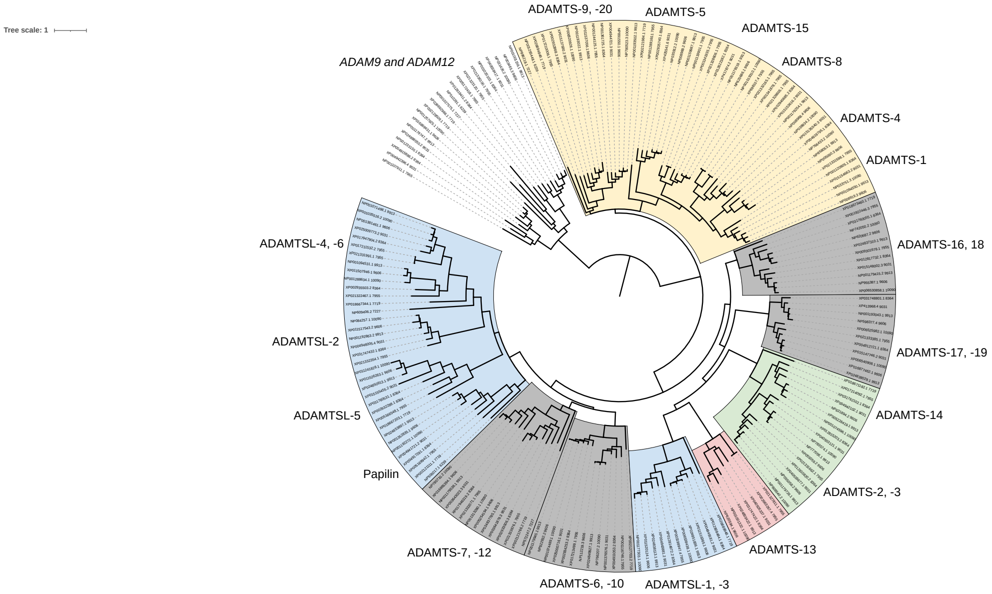

Fig 5. Gene tree with ADAM9 and ADAM12 as the out-group 125 ADAMTS, 48 ADAMTSL, 10 ADAM9 and 11 ADAM12 sequences were used.

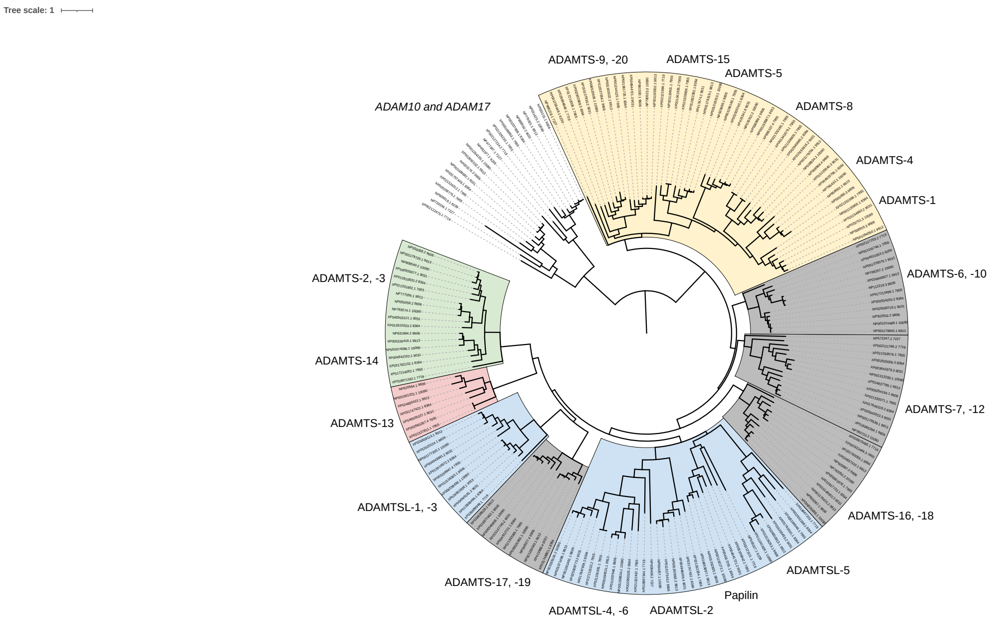

Fig 6. Gene tree with ADAM10 and ADAM17 as the out-group 125 ADAMTS, 48 ADAMTSL, 10 ADAM10 and 11 ADAM17 sequences were used.

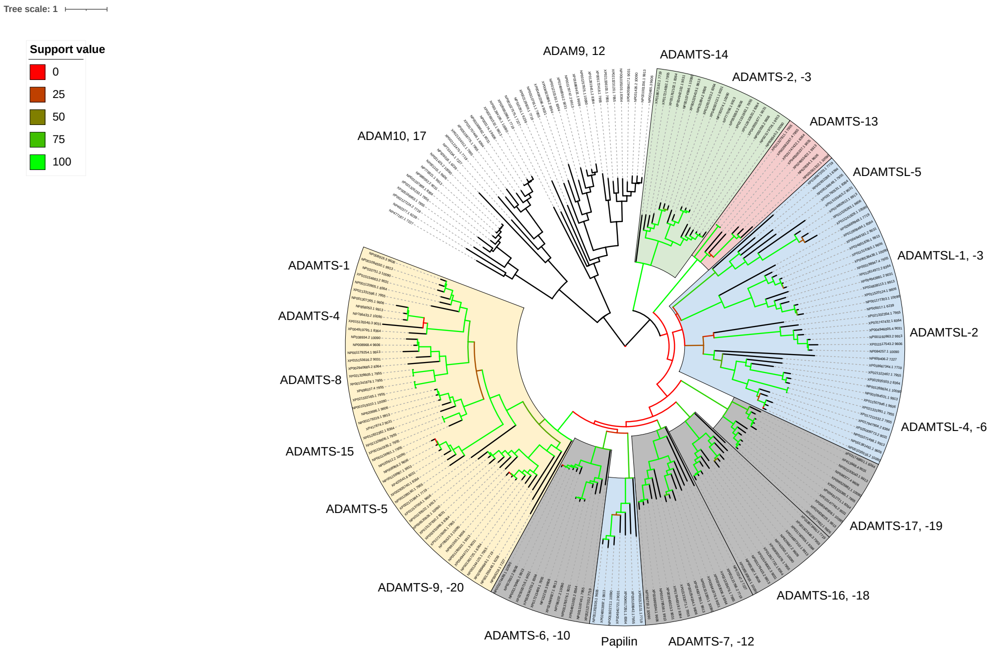

Fig 7. Reference gene tree with support values from the out-group re-sampling trees The out-group gene re-sampling used subsets of the out-group ADAM proteins. We constructed 6 different data-sets, each including all the 125 ADAMTS and 48 ADAMTSL sequences plus a subset of the 41 ADAM out-group sequences including either i) ADAM9, ii) ADAM12, iii) ADAM17, iv) ADAM10, v) ADAM9 plus ADAM12 and vi) ADAM17 plus ADAM10. Then, we constructed the multiple sequence alignment (MSA) and inferred the phylogeny of each data-set. Finally we computed support values on the reference gene tree (branches color scale, from red for 0, to green for 100), using these re-sampled trees.

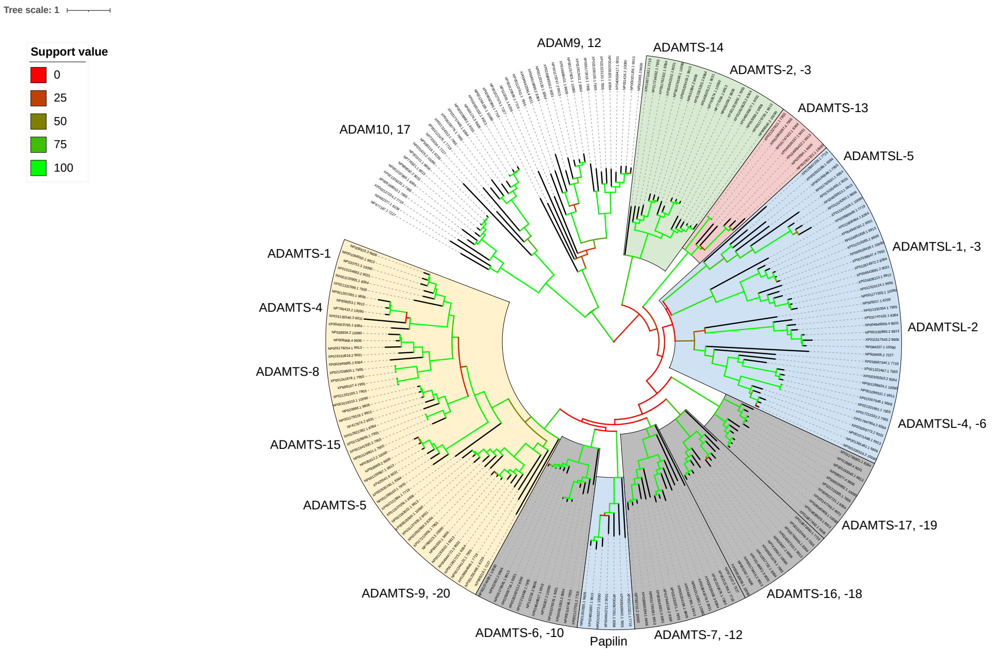

Fig 8. Reference gene tree with support values from the in-group re-sampling trees The in-group gene re-sampling used ADAMTS-TSL proteins from different species. We constructed an alternative ADAMTS-TSL data-set, composed of 255 known sequences (canonical Uniprot) from six species not considered in our reference data-set (*Rattus norvegicus, Xenopus laevis, Canis lupus familiaris, Felis catus, Anolis carolinensis, Pan troglodytes*). Next, we constructed 20 different data-sets, each containing all 173 ADAMTS-TSL sequences from the reference data-set plus 10 sequences randomly chosen from the alternative data-set. Then, we constructed the multiple sequence alignment (MSA) and inferred the phylogeny of each data-set. Finally we computed support values on the reference gene tree (branches color scale, from red for 0, to green for 100), using these re-sampled trees.

References

1. Mirarab S, Nguyen N, Guo S, Wang LS, Kim J, Warnow T. PASTA: Ultra-Large Multiple Sequence Alignment for Nucleotide and Amino-Acid Sequences. Journal of Computational Biology. 2015;22(5):377–386. doi:10.1089/cmb.2014.0156.

2. Stamatakis A. RAxML version 8: a tool for phylogenetic analysis and post-analysis of large phylogenies. Bioinformatics. 2014;30(9):1312–1313. doi:10.1093/bioinformatics/btu033.

3. Wu YC, Rasmussen MD, Bansal MS, Kellis M. TreeFix: Statistically Informed Gene Tree Error Correction Using Species Trees. Systematic Biology. 2013;62(1):110–120. doi:10.1093/sysbio/sys076.

4. Guindon S, Dufayard JF, Lefort V, Anisimova M, Hordijk W, Gascuel O. New Algorithms and Methods to Estimate Maximum-Likelihood Phylogenies: Assessing the Performance of PhyML 3.0. Systematic Biology. 2010;59(3):307–321. doi:10.1093/sysbio/syq010.

5. Letunic I, Bork P. Interactive Tree Of Life (iTOL) v4: recent updates and new developments. Nucleic Acids Research. 2019;47(W1):W256–W259. doi:10.1093/nar/gkz239.
