# Supplementary material for: Phylogenetic inference of the emergence of sequence modules and protein-protein interactions in the ADAMTS-TSL family
Source: PLoS Comput Biol. 2023 Aug 31;19(8):e1011404. doi: 10.1371/journal.pcbi.1011404 (PMC10499240; doi:10.1371/journal.pcbi.1011404)
Supplement: S2 Appendix — (PDF) [file pcbi.1011404.s002.pdf]

Phylogenetic inference of the emergence of sequence modules and protein-protein interactions in the ADAMTS-TSL family

S2 Appendix: **Partial local multiple alignment with Paloma-D**

Olivier Dennler[1,2], François Coste[1], Samuel Blanquart[1], Catherine Belleannée[1], Nathalie Théret[1,2*]

1 Univ Rennes, Inria, CNRS, IRISA, UMR 6074, Rennes, France
2 Univ Rennes, Inserm, EHESP, Irset, UMR S1085, Rennes, France

* nathalie.theret@inserm.fr

We detail here the procedure used to build the partial local multiple alignment (PLMA) [1] of the ADAMTS-TSL and ADAM sequence dataset-214 contained in the fasta file `representatives.fasta` by the command `paloma-d -i representatives.fasta -M 20 -t 10`. An article with a complete presentation of Paloma-D is in preparation, the description will be limited here to this set of options.

Computation of candidate diagonal set. In its initial step, Paloma-D builds a set $\mathcal{C}$ of candidate pairwise local alignments between all the pairs of sequences to align. With the given command line options, $\mathcal{C}$ contains all the diagonals, i.e. gap-free pairs of sequence segments of equal length, considered for alignment by DIALIGN 2 [2] of length smaller or equal to 20 residues (`-M 20`) and of weight greater or equal to 10 (`-t 10`). The weight $w(D)$ of a diagonal D of length l and similarity score s (s is here the sum of the BLOSUM62 [3] scores of the l facing residues in the diagonal) is defined in DIALIGN 2's paper [2] as the negative natural logarithm of the probability $P_2(l,s)$ of finding any diagonal of length l of score at least as large as s somewhere within the comparison matrix of two random sequences of the same length as the original sequences. An advantage of this weight definition is that it enables to compare the similarity of diagonals of different lengths and to keep only those whose similarity is significantly higher than by chance. By default, DIALIGN 2 considers all diagonals of weight greater than 0, which is the expected weight of a random diagonal ($P_2(l,s) = 1$). We use here the more stringent parameters recommended by the Protomata suite for tractable and safe identification of strongly conserved domains [4]. Indeed, the recommended weight threshold of 10 keeps only strongly conserved diagonals. This is the maximal weight thresholds recommended by the Protomata suite, corresponding to probabilities $P_2(l,s)$ lower than approx. 5×10^{-5}. For comparison, other thresholds recommended by the Protomata suite are 1, 3 and 5 corresponding respectively to $P_2(l,s)$ values lower than approx. 4×10^{-1}, 5×10^{-2}, 6×10^{-3}. The maximal length parameter is not as critical since it does not limit the size of identifiable domains since, thanks to the local extension introduced below, the diagonals can be chained to identify conservations longer than the lengths of diagonals used. We kept here the recommended value of 20 residues that was experimentally observed to provide a good signal/tractability trade-off for the identification of domains in the Protomata suite.

Selection and integration of diagonals. The core loop of Paloma-D integrates iteratively compatible diagonals from $\mathcal{C}$ to the set of PLMA local alignments $\mathcal{L}$ which is here a set of diagonals. With the given options, each candidate diagonal from $\mathcal{C}$ is added in turn to $\mathcal{L}$, as well as the diagonals induced by transitivity and extension of local alignments, if and only if the induced alignment is consistent [5]. Otherwise, the candidate is incompatible with previously added diagonals and is discarded. Let us specify which are the induced diagonals with the help of Fig 1. The transitive closure of the local alignments consists in adding automatically the diagonal $T = (o1, o3)$ to $\mathcal{L}$ whenever there exists $D = (d1, d2)$ and $E = (e2, e3)$ in $\mathcal{L}$ such that $d2$ and $e2$ overlap on a segment $o2$ aligned to $o1$ by D and to $o3$ by E. This operation performs the consistent closure of pairwise local alignments to multiple sequences. Note however

that $(o1, o3)$ is not required to belong to $\mathcal{C}$ since the experimental permissive mode of Paloma is now activated by default in Paloma-D to fasten the search. The extension of local alignment, for its part, enables longer local alignments to be built from smaller ones: it consists in replacing any pair of diagonals $U = (u1o1, u2o2)$ and $V = (o1v1, o2v2)$ from $\mathcal{L}$, with a segment overlap $o1$ on one sequence and $o2$ on the other sequence, by the diagonal $L = (u1o1v1, u2o2v2)$ covering them.

In this greedy approach, the ordering of the candidates is important to establish a preference between them. In Paloma-D, the default ordering aims at favoring diagonals whose segments are conserved at most in most of the sequences. It can also be seen as a heuristic aiming at maximizing the final alignment score, defined here as the sum of weights of the diagonals in $\mathcal{L}$. More precisely, let us denote by $\mathcal{X}$ the set of sequences to align and by Segments(S) the set of segments of a sequence S. For each diagonal D in $\mathcal{C}$ aligning two segments d_1 and d_2, we define for each sequence S:

$$d^S_{max}(d_1, d_2) = \underset{\{d \in \text{Segments}(S) \setminus \{d_1, d_2\} :\ (d_1,d) \in \mathcal{C}, (d_2,d) \in \mathcal{C}\}}{\text{argmax}} \left(w(d_1, d) + w(d_2, d) \right).$$

If the set $d^S_{max}(d_1, d_2)$ is not empty, its elements are each a segment of the sequence S that would maximize the sum of the weights if aligned to d_1 and d_2 with diagonals from $\mathcal{C}$. We denote by $d^S_{max}(d_1, d_2)[1]$ the first element of this set with respect to an arbitrary order. Considering all the sequences, we can now compute the maximal sum of weights contribution of (d_1, d_2) to the score of a local alignment extending it optimally to the other sequences with diagonals pairs of $\mathcal{C}$ by:

$$W_{max}(d_1, d_2) = w(d_1, d_2) + \sum_{S \in \mathcal{X} :\ d^S_{max}(d_1,d_2) \neq \emptyset} w(d_1, d^S_{max}(d_1, d_2)[1]) + w(d_2, d^S_{max}(d_1, d_2)[1]).$$

$W_{max}(d_1, d_2)$ is an estimation based on $\mathcal{C}$ of the contribution that (d_1, d_2) can make to the global score of $\mathcal{L}$. It is the default heuristic score used in Paloma-D to sort by decreasing values the diagonals from $\mathcal{C}$ considered for addition in $\mathcal{L}$.

Alignment blocks. Once the set of diagonals $\mathcal{L}$ is built, the final step consists in identifying the set of the alignment blocks $\mathcal{B}$ which are the maximal multiple gapless alignment units induced by $\mathcal{L}$, aligning each a set of segments conserved in, and only in, a specific subset of sequences from $\mathcal{X}$. An alignment block can be formally defined here as a set of aligned segments such that 1) the block aligns each pair of its segments on their full length and this alignment is a restriction to these segments of a diagonal from $\mathcal{L}$ 2) there exists no diagonal in $\mathcal{L}$ aligning a position of a segment of the block to another position than those to which the position is aligned in the block 3) the set of aligned segments is maximal in the sense that there exists no other set of aligned segments including it that satisfies the two previous conditions.

The different steps of this alignment procedure are summarized by Algorithm 1 and an example of partial local multiple alignment graph showing the alignment blocks built from a set of candidate diagonals on the sequences is presented in Fig 2.

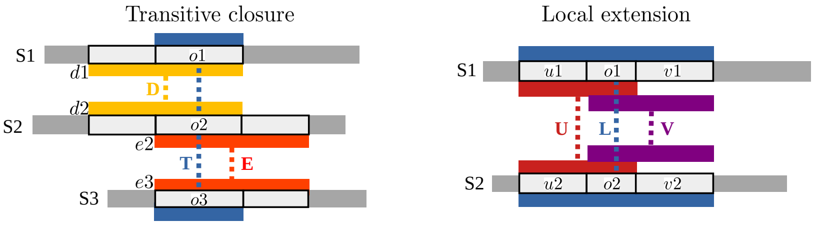

Fig 1. Induced diagonals. Left: If diagonals D and E overlap on a segment $o2$ which is aligned to a segment $o1$ by D and to a segment $o3$ by E, then the diagonal $T = (o1, o3)$ is added to $\mathcal{L}$ by the transitive closure of diagonals. Right: If diagonals U and V overlap on the segments o_1 and o_2, it implies a local alignment L covering both diagonals which is added to $\mathcal{L}$ in replacement of U and V by the local extension of diagonals.

Algorithm 1 Build partial local multiple alignment

Input: set of sequences $\mathcal{X}$
Segments $\leftarrow \{(S, b, e) \colon S \in \mathcal{X}, 1 \leq b \leq e \leq |S|, e - b + 1 \leq 20\}$ ▷ 20 is the chosen maximal length
$\mathcal{C} \leftarrow \{(d_1, d_2) \in$ Segments $\times$ Segments$\colon w(d_1, d_2) \geq 10\}$ ▷ 10 is the chosen weight threshold
$\mathcal{L} \leftarrow \emptyset$
for $(d_1, d_2) \in \mathcal{C}$ by decreasing value of $W_{max}(d_1, d_2)$ **do**
    if $\mathcal{L} \cup \{(d_1, d_2)\}$ induces a consistent alignment **then** ▷ Compatibility test
        $\mathcal{L} \leftarrow$ transitive closure and local extension of $\mathcal{L} \cup \{(d_1, d_2)\}$
    end if
end for
$\mathcal{B} \leftarrow$ alignment blocks defined by $\mathcal{L}$ on $\mathcal{X}$
return $(\mathcal{L}, \mathcal{B})$

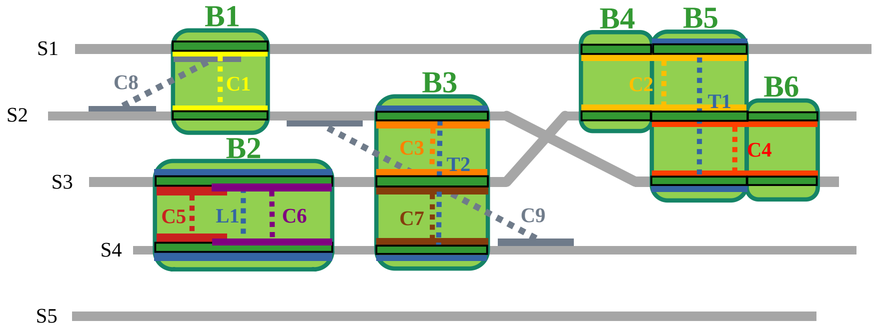

Fig 2. Partial local multiple alignment built from a set of diagonals. In this schematic example, S1. . . S5 are the sequences to align, C1. . . C7 are the candidate diagonals successfully added by Paloma-D (examples of discarded diagonals C8 and C9, respectively incompatible with C1 and with {C3,C7}, are also shown), T1 and T2 are the diagonals added by transitive closure of respectively {C2,C4} and {C3,C7}, L1 is the diagonal replacing C5 and C6 by local extension and B1. . . B6 are the resulting alignment blocks. We see here that blocks can be shorter than the candidate diagonals (see for instance B5) or longer (see for instance B2).

References

1. Kerbellec G. Apprentissage d'automates modélisant des familles de séquences protéiques. (Learning automata modelling families of protein sequences) [PhD Thesis]. University of Rennes 1, France; 2008. Available from: https://tel.archives-ouvertes.fr/tel-00327938.

2. Morgenstern B. DIALIGN 2: improvement of the segment-to-segment approach to multiple sequence alignment. Bioinform. 1999;15(3):211–218. doi:10.1093/bioinformatics/15.3.211.

3. Henikoff S, Henikoff JG. Amino acid substitution matrices from protein blocks. Proceedings of the National Academy of Sciences. 1992;89(22):10915–10919.

4. Protomata 2.0;. http://protomata-learner.genouest.org.

5. Abdeddaïm S, Morgenstern B. Speeding Up the DIALIGN Multiple Alignment Program by Using the 'Greedy Alignment of BIOlogical Sequences LIBrary' (GABIOS-LIB). In: Gascuel O, Sagot MF, editors. Computational Biology. Berlin, Heidelberg: Springer Berlin Heidelberg; 2001. p. 1–11.
