# Supplementary material for: Phylogenetic inference of the emergence of sequence modules and protein-protein interactions in the ADAMTS-TSL family
Source: PLoS Comput Biol. 2023 Aug 31;19(8):e1011404. doi: 10.1371/journal.pcbi.1011404 (PMC10499240; doi:10.1371/journal.pcbi.1011404)
Supplement: S3 Appendix — Itol tree screenshots and usage examples. (PDF) [file pcbi.1011404.s003.pdf]

Phylogenetic inference of the emergence of sequence modules and protein-protein interactions in the ADAMTS-TSL family

S3 Appendix: **Browsing the ADAMTS-TSL Itol tree**

Olivier Dennler[1,2], François Coste[1], Samuel Blanquart[1], Catherine Belleannée[1], Nathalie Théret[1,2*]

1 Univ Rennes, Inria, CNRS, IRISA, UMR 6074, Rennes, France
2 Univ Rennes, Inserm, EHESP, Irset, UMR S1085, Rennes, France

* nathalie.theret@inserm.fr

1 Navigating the tree

An important contribution of this work is the availability of all the data through an interactive tree (automatically generated at the end of the pipeline), using the Interactive Tree Of Life software, Itol [1]. The ADAMTS-TSL Itol tree is available Here. When accessing the Itol tree, the user will be presented with the original view of our ADAMTS-TSL tree (Fig 1), allowing him to navigate in the tree, to modify the representation, or to activate different datasets. Pruning the tree allows working on only relevant parts of the tree, thereby reducing possible delays due to the volume of data (`https://itol.embl.de/help.cgi#prune`).

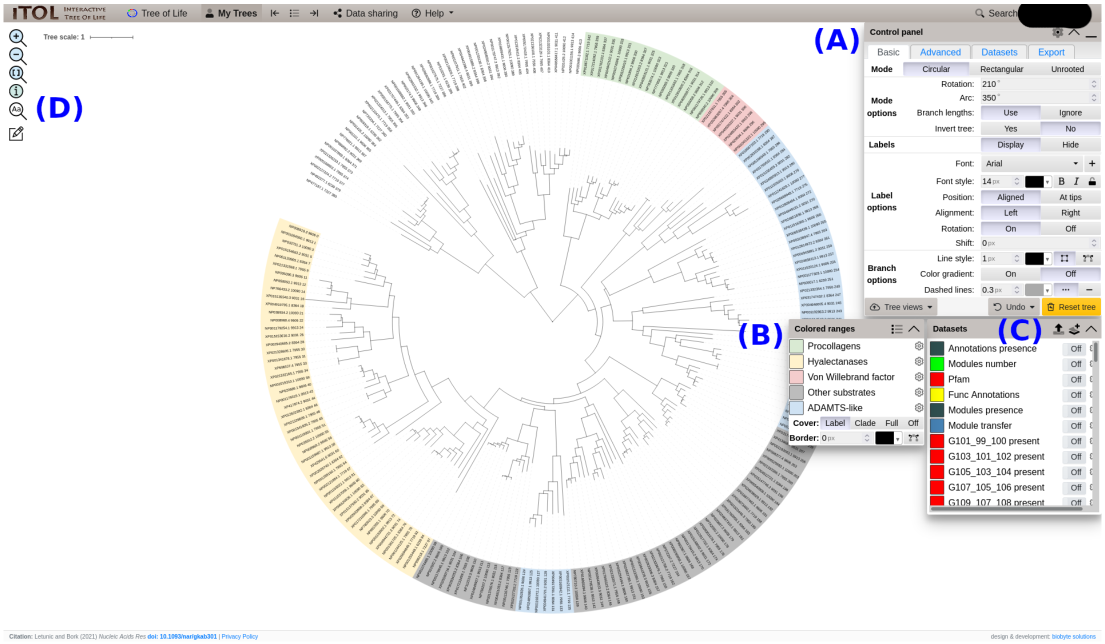

Fig 1. Original view of the ADAMTS-TSL Itol tree
(A) Itol control panel, (B) Colored ranges panel, (C) Datasets activation panel, and (D) Search tree node engine.

An HTML popup window (Fig 2) provides detailed information about each gene node, including the name of the gene, the nature of the gene (ancestor or leaf), the Protein-Protein Interactions (PPIs) associated with the gene and PPI gain/loss with respect to the ancestor, the module composition of the gene and module gain/loss with respect to the ancestor.

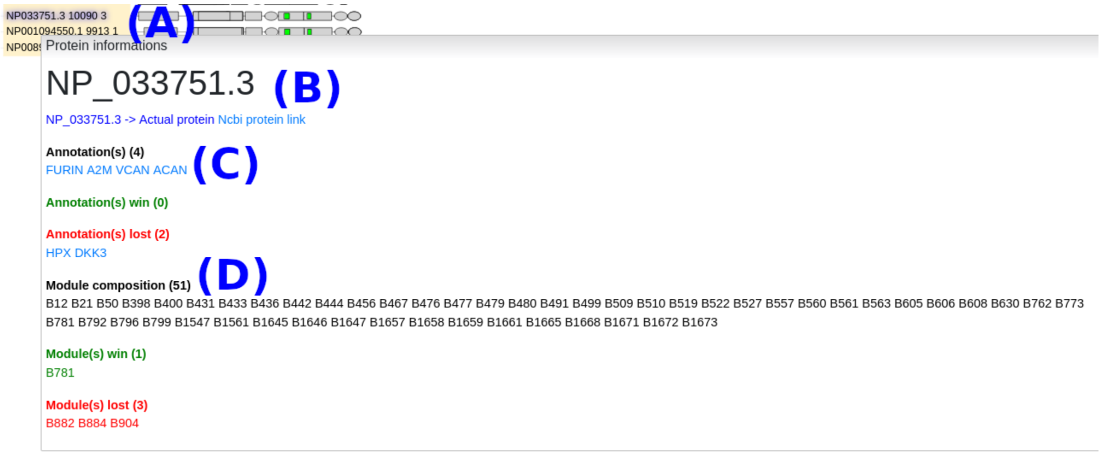

Fig 2. Node popup

(A) Each gene node (ancestral or leaf) has a custom popup containing all information about protein, module(s) and PPI(s). (B) Protein information : node name (with RefSeq ID for leaves) and link to the protein entry on the NCBI website. (C) List of annotations (here PPI) present, gain and lost at this gene node. (D) List of modules present, gain and lost at this gene node.

Saved views (Fig 3) allow quick access to visualizations made beforehand to study cases of interest (e.g., the study cases of the article).

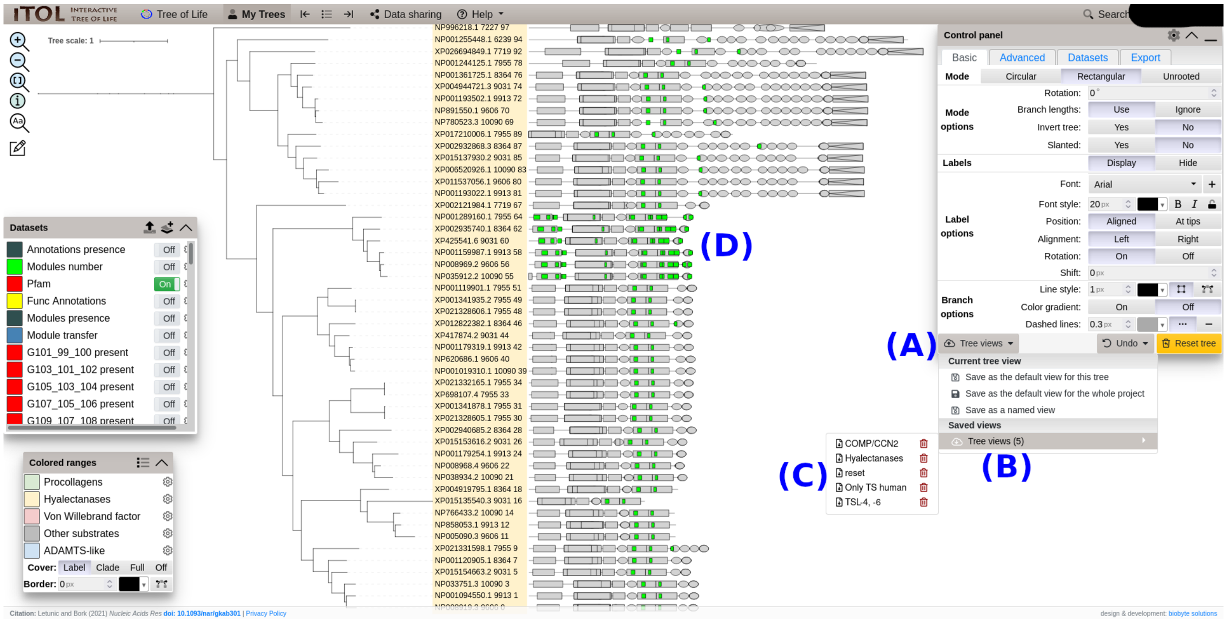

Fig 3. Saved views

(A) Tree views panel. (B) Saved views / Tree views panel. (C) List of our customs views (e.g., the hyalectanases pruned subtree and the corresponding modules signatures). (D) The hyalectanases saved view.

2 Datasets usage

The Itol tree also provides annotations as datasets, including the number, the composition and the transfer of modules, the domain composition, the speciation events and the presence of PPI (Func Annotation). Fig 4 illustrates the ”Module number” and the ”Func Annotations” datasets.

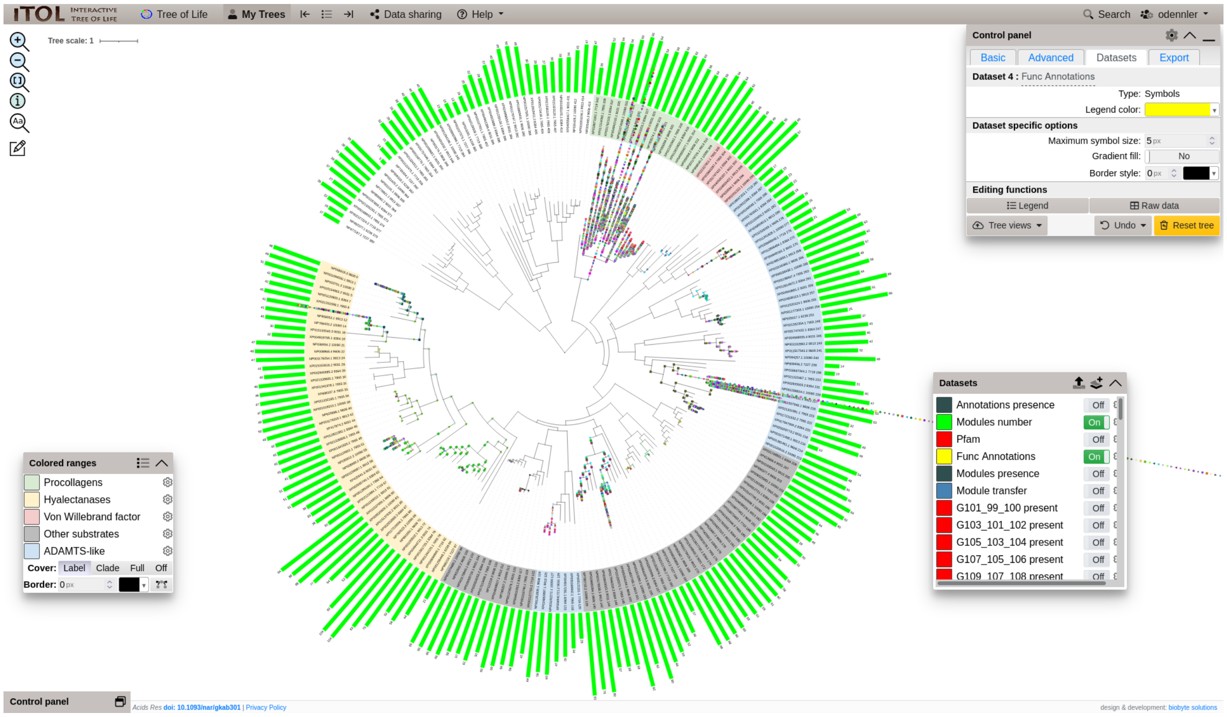

Fig 4. Example of datasets
Both the ”Module number” (number of modules at each leaf) and ”Func Annotations” (PPI presences are symbolized with shape/color combinations) datasets are enabled on the default view.

In particular, the ”Module composition” and the ”Pfam” datasets visualize module (Fig 5) and domain (Fig 6) composition for each leaf, with all module/domain information available on popups.

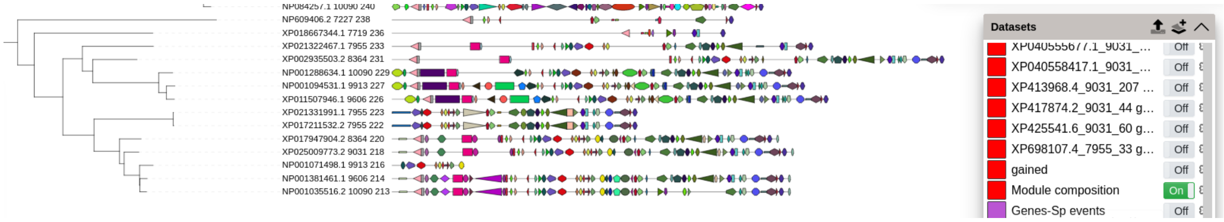

Fig 5. Module composition dataset
All leaf module compositions are represented by a mosaic of modules. Each module is a combination of shape/color and has a popup with its name and position on the protein sequence.

Each gene has its own module signature annotation (1 dataset per gene node) for both present (Fig 7) and gained (Fig 8) modules. All module signatures can be visualized with their domain contexts by first enabling the domain composition annotations (Pfam dataset).

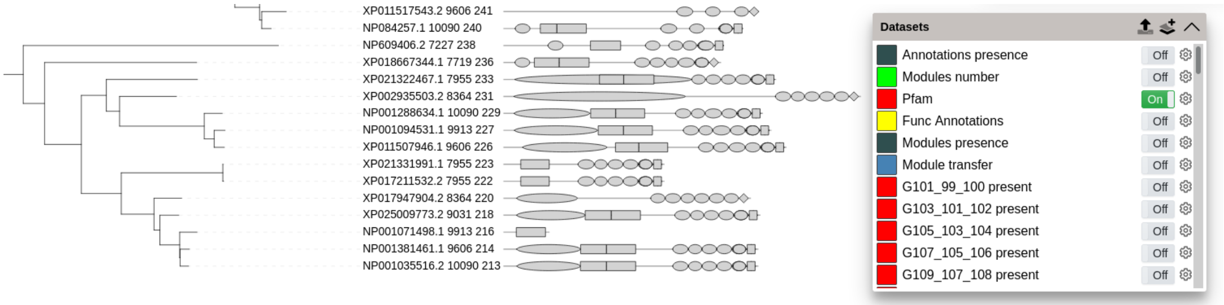

Fig 6. Pfam dataset
All leaf domain compositions are represented by grey shapes. Each domain has a popup with its ID and position on the protein sequence.

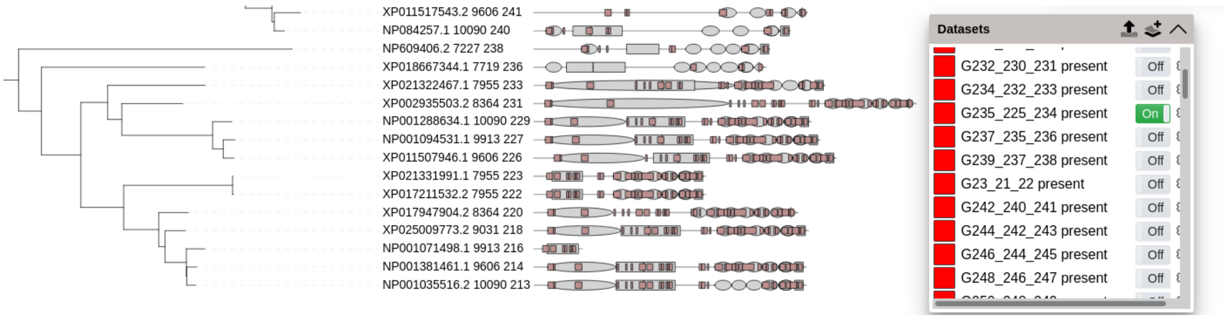

Fig 7. Example of ancestral module presence dataset
The "Pfam dataset" has been enabled prior to the "G235_225_224_present" dataset. All modules present (module composition) at the G235 ancestral gene node are represented as brown boxes on the actual proteins (leaves) where they are present.

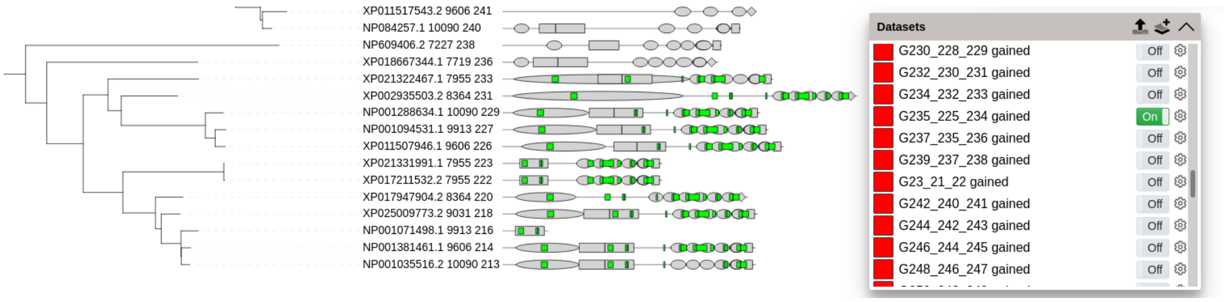

Fig 8. Example of ancestral module gained dataset
The "Pfam dataset" has been enabled prior to the "G235_225_224_gained" dataset. All modules gained (present but absent in its ancestor) at the G235 ancestral gene node are represented as green boxes on the actual proteins (leaves) where they are present.

References

1. Letunic I, Bork P. Interactive Tree Of Life (iTOL) v4: recent updates and new developments. Nucleic Acids Research. 2019;47(W1):W256–W259. doi:10.1093/nar/gkz239.
