# Supplementary figures and images for: Phylogenetic inference of the emergence of sequence modules and protein-protein interactions in the ADAMTS-TSL family
Source: PLoS Comput Biol. 2023 Aug 31;19(8):e1011404. doi: 10.1371/journal.pcbi.1011404 (PMC10499240; doi:10.1371/journal.pcbi.1011404)

**(A) pLDDT**

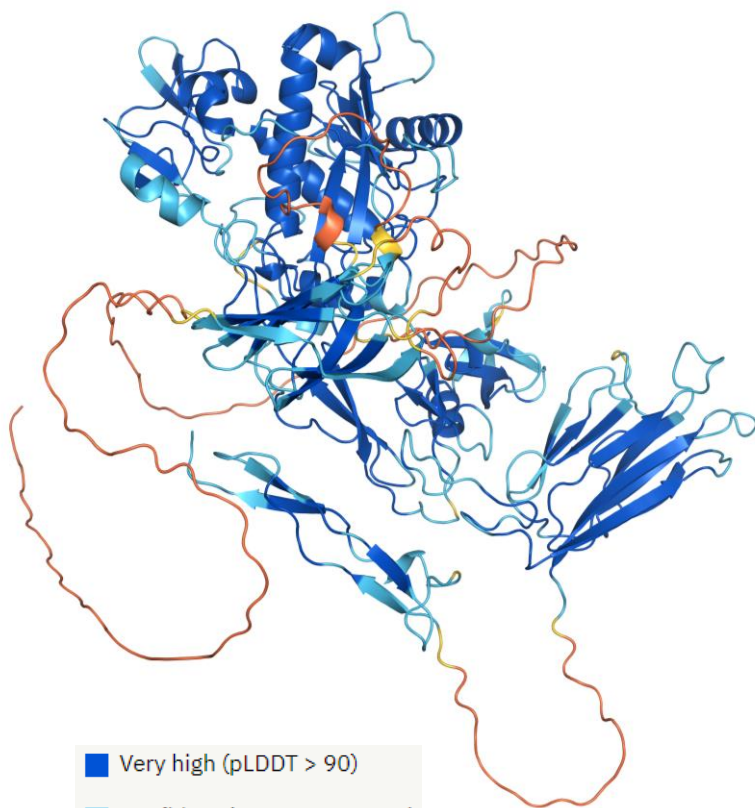

- Very high (pLDDT > 90)
- Confident (90 > pLDDT > 70)
- Low (70 > pLDDT > 50)
- Very low (pLDDT < 50)

**(B) PAE**

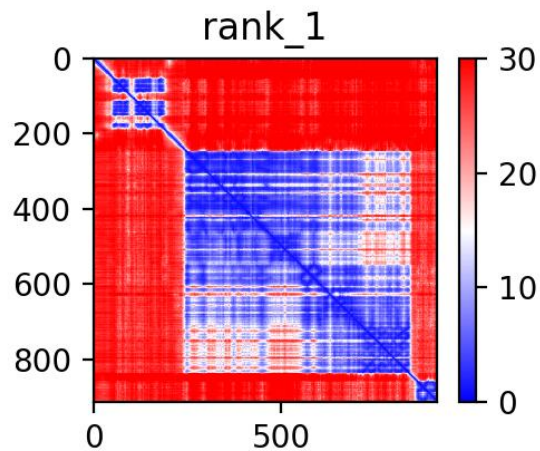

Supplement: S1 Fig — (PDF) [file pcbi.1011404.s013.pdf]

(A) pLDDT

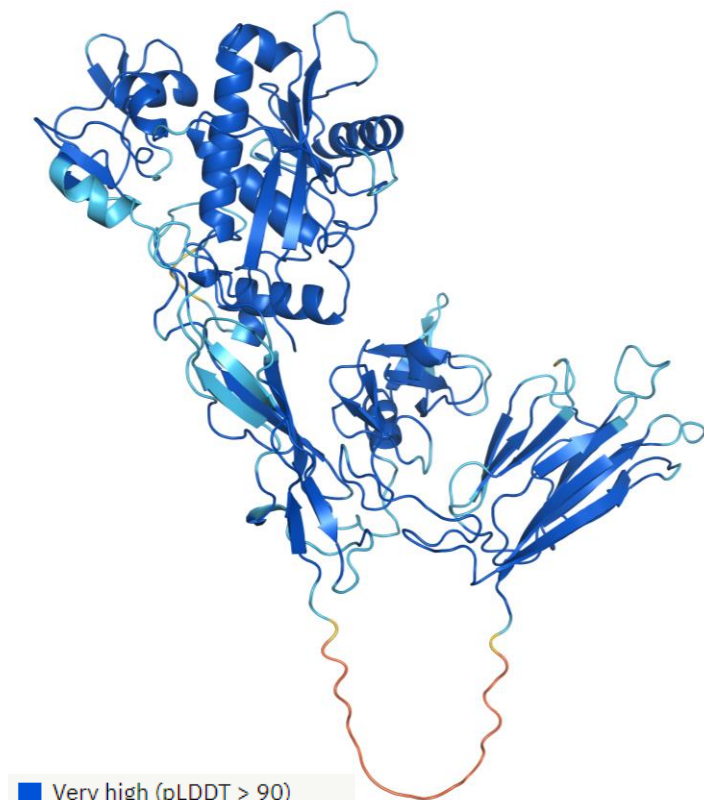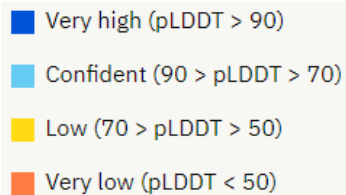

(B) PAE

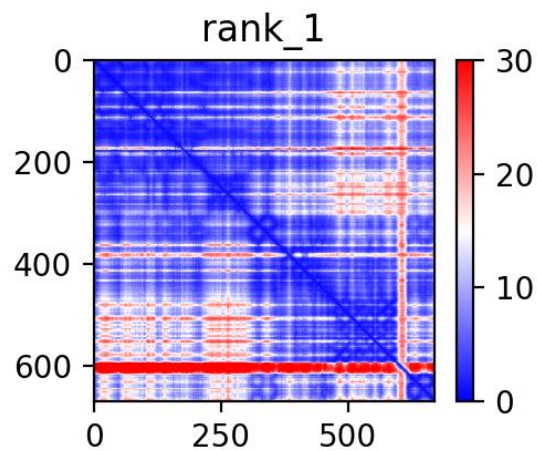

Supplement: S2 Fig — (PDF) [file pcbi.1011404.s014.pdf]
